# Efficacy and safety of IL-23 p19 inhibitors in the treatment for inflammatory bowel disease: a systematic review and meta-analysis

**DOI:** 10.3389/fphar.2025.1490667

**Published:** 2025-04-28

**Authors:** Shuhan Wang, Hui Sun, Qian Wang, Han Xiao

**Affiliations:** Lanzhou University Second Hospital, Lanzhou University, Lanzhou, China

**Keywords:** IL-23 p19 inhibitors, inflammatory bowel disease, IBD pharmacotherapy, remission, adverse events

## Abstract

**Background:**

The treatment outcomes of inflammatory bowel disease (IBD) have been significantly improved by the advent of new biologics, including ulcerative colitis (UC) and Crohn’s disease (CD), particularly for refractory cases. However, the growing number of therapeutic options has also complicated clinical decision-making regarding drug selection and switching. The overall performance of IL-23 p19 inhibitors for the treatment of IBD was evaluated by the systematic review and meta-analysis in this study.

**Objective:**

The objective of this study was to combine the multiple indicators to accurately evaluate the efficacy and safety of IL-23 p19 inhibitors, aimed to offer an insight into the development of clinical physicians’ medication.

**Methods:**

A comprehensive literature review on PubMed, Embase, Web of Science, and Cochrane Library until June 2024 was conducted in this study, which mainly focused on the randomized controlled trials (RCTs) to evaluate the IL-23 p19 inhibitors within adult patients with UC or CD. Additionally, the clinical outcomes, endoscopic findings, histological assessments, and safety profiles were aggregated and subjected to analysis by a random-effects model.

**Results:**

Twenty-five RCTs [15 CD, 10 UC] were involved in this study, and it was revealed that IL-23 p19 inhibitors showed significant effects on clinical remission (CR) in IBD, regardless of induction or maintenance treatment (CD, induction: risk ratio [RR] 1.95, 95% confidence interval [CI] 1.71–2.23; I^2^ = 0%, p = 0.68; UC, induction: RR 2.69, 95% CI 1.80–4.03; I^2^ = 50%, p = 0.09; CD, maintenance: RR 1.24, 95% CI 1.04–1.48; I^2^ = 0%, p = 0.57; UC, maintenance: RR 2.62, 95% CI 0.92–7.49; I^2^ = 42%, p = 0.19), and the risk of adverse events (AEs) was similar to that of placebo (CD, induction: RR 0.88, 95% CI 0.82–0.94; I^2^ = 2%, p = 0.41; UC, induction: RR 0.92, 95% CI 0.82–1.03; I^2^ = 0%, p = 0.54; CD, maintenance: RR 1.00, 95% CI 0.89–1.13; I^2^ = 29%, p = 0.25; UC, maintenance: RR 0.96, 95% CI 0.87–1.06; I^2^ = 0%, p = 0.44).

**Conclusion:**

In IBD treatment, IL-23 p19 inhibitor therapy exhibited effective functions in the inducement and maintenance of clinical and endoscopic remissions, as well as in some histological cases.

**Systematic Review Registration:**

https://www.crd.york.ac.uk/PROSPERO/view/CRD42024569807, identifier CRD42024569807

## 1 Introduction

Since the 21st century, the shifts in dietary habits and rising stress levels have contributed to a significantly increasing trend of IBD globally (Zhou et al., 2023). Notably, IBD followed a four-phase epidemiological trajectory: Emergence, Accelerated Incidence, Compounding Prevalence, and Prevalence Equilibrium. Additionally, developing countries remained in the emergence phase in 2020, while Western nations entered the compounding prevalence phase, in which the prevalence was expected to show a stable trend under the background of aging populations and rising mortality rates (Kaplan and Windsor, 2021). In the U.S. (Lewis et al., 2023), 0.7% of the population (∼2.39 million people) were diagnosed with IBD, and it was found that there were significant racial disparities in prevalence per 100,000: White 812 (95%CI 802–823), Black 504 (482–526), Asian 403 (373–433), Hispanic 458 (440–476). It was underscored by the trends mentioned above that enhanced prevention, individualized therapies, and health system adjustments were in urgent need for an aging IBD population. In addition, it was also emphasized by these epidemiological trends that the improvement of prevention strategies, personalized treatment approaches, and adaptations in healthcare systems was necessary to meet the growing demands of an aging IBD population. Besides, IBD was composed of CD and UC, and it was featured by chronic progressive or relapsing inflammation of the gastrointestinal tract (Piovani et al., 2019). Furthermore, the high cost of long-term management of IBD tended to result in a substantial economic burden for patients, their families, and the healthcare systems. Although the exact etiology of IBD required further studies, it was revealed that genetic susceptibility, environmental factors, and immune system dysregulation showed critical functions in its pathogenesis (Kotla and Rochev, 2023).

Conventional treatments for IBD were typically composed of aminosalicylates, corticosteroids, and immunosuppressive agents (Cai et al., 2021). Nevertheless, the prolonged application of these agents tended to result in serious side effects and relapse, and many patients failed to respond adequately to the treatment (Luo et al., 2022). I Therefore, biological therapies have gained more attention in recent years as a promising and effective approach to the management of IBD (Cheifetz et al., 2021). Additionally, these agents showed their functions based on the inhibition of the key cytokines involved in the inflammatory cascade, which was beneficial to control the disease activity and sustain remission (Liu et al., 2023). Over the last decade, biologics—particularly tumor necrosis factor-alpha (TNF-α) inhibitors—have been widely applied in IBD (Souza et al., 2023). However, up to 30% of patients exhibited primary nonresponse, while another 23%–46% developed secondary loss of response over time (Roda et al., 2016). In addition, TNF-α inhibitors were confirmed to be connected with the increased risks of infections, including tuberculosis, varicella, non-melanoma skin cancers, and non-Hodgkin lymphoma (Calip et al., 2018). Overall, it was underscored by these safety concerns to develop novel therapeutic alternatives with improved efficacy and tolerability.

IL-23 was considered a heterodimeric cytokine consisting of the p40 and p19 subunits, and it shared the p40 subunit with IL-12, which was stabilized by disulfide bonds (Lupardus and Garcia, 2008). Additionally, the p19 subunit was initially identified by Oppmann et al. (2000), which belonged to the IL-6 cytokine family and represented a novel cytokine component. Besides, IL-23 could facilitate the expansion and maintenance of Th17 cells (Schmitt et al., 2021), which were referred to as the major contributors to inflammation by inducing the production of pro-inflammatory cytokines including IL-17, IL-21, and IL-22. Notably, these cytokines exhibited essential functions in host defense against bacterial and fungal pathogens, and they were recognized as critical mediators of autoimmune processes (Saxton et al., 2023). Additionally, these cytokines exhibited significant effects on IBD by driving aberrant immune responses and promoting intestinal tissue injury (Saez et al., 2023). It was demonstrated by animal studies that the mice deficient in IL-23 or its receptor exhibited pronounced anti-inflammatory functions in experimental colitis models, which could enhance the pivotal functions of IL-23 in IBD pathogenesis (Yen et al., 2006). Besides, it was further indicated by the clinical evidence that IL-23 levels were markedly elevated within both serum and intestinal tissues of IBD patients, which showed strong connections with the disease activity (Kvedaraite et al., 2016).

Due to the central functions in the activation of pro-inflammatory cytokine release, the IL-23 signaling pathway has become a key therapeutic target in IBD. Additionally, IL-23 p19 inhibitors were analyzed in clinical trials and observational studies due to their efficacy in various immune-mediated diseases, such as psoriasis and ankylosing spondylitis (Reich et al., 2019; Baeten et al., 2018). Nevertheless, their clinical efficacy in IBD remained to be systematically assessed and clarified in the future. Given the heterogeneity across current clinical trial outcomes, a meta-analysis was required for the integration of the available evidence and clear insights into the therapeutic values of IL-23 p19 inhibitors in IBD. Besides, a thorough understanding of the clinical efficacy could provide evidence-based treatment decisions for IBD, alongside the support for the development of drug and personalized therapeutic strategies. At present, multiple phase II and III clinical trials of IL-23 p19 antagonists for IBD were underway. Overall, this study aimed to assess the efficacy and safety of e IL-23 p19 antagonists in IBD through an evidence-based approach.

## 2 Methods

This research was prospectively registered with PROSPERO (CRD42024569807), and all procedures were strictly following the Cochrane Handbook for Systematic Reviews of Interventions.

### 2.1 Data sources

A literature review on PubMed, Embase, Web of Science, and Cochrane Library until June 2024 was conducted based on predefined search terms. Additionally, relevant conference proceedings were reviewed to identify eligible abstracts. Besides, titles, abstracts, and full texts were screened by two independent reviewers to select the studies that were in agreement with the predefined inclusion and exclusion criteria. The complete PubMed search strategy was provided in Supplementary Data.

### 2.2 Inclusion and study selection

The inclusion criteria were listed below (Zhou et al., 2023): clinical randomized controlled trials (RCTs) (Kaplan and Windsor, 2021); phase II or III double-blind, the evaluation of IL-23 p19 inhibitors in IBD by placebo-controlled trials (Lewis et al., 2023); enrolled adult patients of any race or gender diagnosed with moderate to severe IBD based on the Fifth Consensus Guidelines for IBD diagnosis and treatment. For CD, disease severity was defined by the Crohn’s Disease Activity Index (CDAI): scores from 150 to 220 were considered mild, 221–450 were moderate, and >450 was severe. For UC, severity was assessed by the modified Mayo score: ≤4 (with individual subscores ≤1) indicated mild disease, 5–6 moderate, and 7–12 represented severe (Piovani et al., 2019); The primary outcome was the CR rate (Kotla and Rochev, 2023); Regarding the treatment group, IL-23 p19 inhibitors were administered at various dosages and via different routes according to the included studies, without predefined upper or lower limits during the selection (Cai et al., 2021); A history of treatment with conventional therapies was not considered an exclusion criterion, including glucocorticoids, immunomodulators, or other biologics (Luo et al., 2022); Patients who received conventional therapies at baseline were required to maintain stable doses during this research, including oral mesalamine compounds, corticosteroids, and immunomodulators (azathioprine or 6-mercaptopurine). The application of these concomitant medications was permitted to minimize the disease fluctuations and prevent the flare-ups caused by abrupt discontinuation. Notably, the dose escalation was not allowed during the trial. Additionally, the secondary outcomes were mainly composed of the clinical response rate, endoscopic and histological assessments, alongside the incidence of treatment-emergent adverse events. Definitions of all outcome measures were detailed in Supplementary Tables S2,S3.

The exclusion criteria were listed below: non- RCT studies, reviews, duplicate publications, studies without a control group or lacking placebo in the control group, studies with different primary outcome measures, studies involving pediatric or pregnant populations, studies involving IBD patients with other serious comorbidities, alongside the studies with methodological flaws or incomplete data.

### 2.3 Outcomes

Regarding the induction trials, the primary endpoint was the proportion of patients achieving CR. Regarding the maintenance trials, it mainly analyzed the remission maintaining proportion among those with the initial response to the relevant treatment. Additionally, CR was typically defined by the standardized disease activity indices. Besides, the secondary endpoints were mainly composed of the patient’s proportion with a clinical response; changes in stool frequency and abdominal pain scores (SF/APS); endoscopic response and mucosal healing; and histologic response and remission. Additionally, safety outcomes were also assessed, such as AEs, serious adverse events (SAEs), and treatment discontinuation due to the relevantly dangerous situation. Besides, AEs were coded with the Medical Dictionary for Regulatory Activities and graded with the National Cancer Institute Common Terminology Criteria for Adverse Events.

### 2.4 Risk of bias assessment

The risk of bias assessment followed a rigorous, structured process to ensure objectivity and accuracy. Additionally, a dual-reviewer approach was employed in the minimization of subjective interpretation, and any disagreements were handled by discussion or the participation of a third reviewer. The assessment was conducted by the Cochrane Risk of Bias 2 (RoB 2) tool, which could evaluate six domains (Zhou et al., 2023): random sequence generation (selection bias) (Kaplan and Windsor, 2021), allocation concealment (selection bias) (Lewis et al., 2023), blinding of participants and personnel (performance bias) (Piovani et al., 2019), blinding of outcome assessment (detection bias) (Kotla and Rochev, 2023), incomplete outcome data (attrition bias), and (Cai et al., 2021) selective reporting (reporting bias). Additionally, each study was ranked as having a low, high, or unclear risk of bias. The structured framework of the RoB 2 tool could enhance the reliability of the evaluation by providing a consistent and transparent methodology. Besides, a strong commitment to methodological integrity could be reflected by this rigorous approach, which contributed to the overall credibility of the findings.

### 2.5 Data analysis

A random-effects model was employed in the explanation of variability, which could obtain a more comprehensive and nuanced synthesis of the evidence. This approach was particularly appropriate when the assumption of homogeneity across studies may not hold. The DerSimonian and Laird method was employed in the accommodation of potential variations in effect sizes, which could enhance the robustness and generalizability of the meta-analysis. An intention-to-treat (ITT) analysis was adopted to provide a realistic estimate of treatment efficacy, in which the incomplete follow-up data was taken into account. Additionally, the risk ratios (RRs) and 95% confidence intervals (CIs) were calculated in this study. The statistical heterogeneity was assessed by the I^2^ statistic, and the values of 0%–25%, 25%–50%, 50%–75%, and 75%–100% could indicate low, moderate, substantial, and considerable heterogeneity, respectively. Moreover, subgroup analysis was employed in the exploration of the sources of heterogeneity, and a p-value <0.10 was considered to show statistically significant heterogeneity. All the statistical analyses were operated by the Review Manager (version 5.3).

## 3 Results

### 3.1 Selection process

**FIGURE 1:**
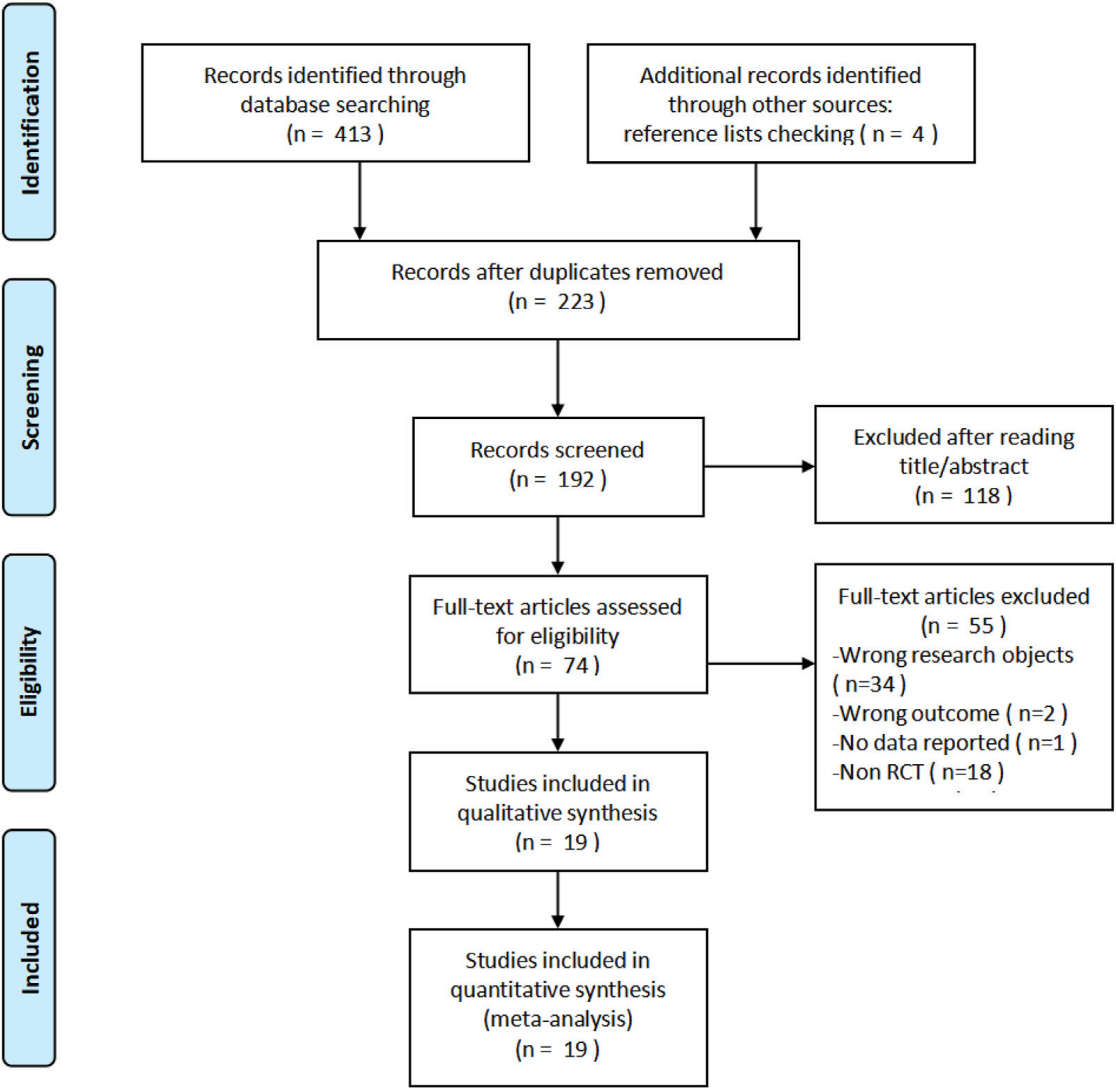
PRISMA flow diagram of study selection. A total of 417 records were identified, and after screening and eligibility assessment, 19 studies were included in the meta-analysis.

During this research, 417 records were identified, which were composed of 413 from database searches and four additional records from the screening of the reference list. After the removal of 223 duplicates, 192 unique records remained for the title and abstract screening, among which 118 were excluded based on the irrelevance and inclusion criteria. Additionally, 55 of the remaining 74 studies were excluded for the following reasons based on the full-text assessment: irrelevant study populations (n = 34), inappropriate outcome measures (n = 2), lack of available data (n = 1), and non-randomized controlled trial (non-RCT) design (n = 18). Ultimately, 19 articles were included in this study (D'Haens et al., 2022; Ferrante et al., 2022; Feagan et al., 2017; Gao et al., 2024; D'Haens G. R. et al., 2021; Panaccione et al., 2021; Sands et al., 2022; Magro et al., 2023a; Pai et al., 2021; Sands et al., 2017; Sandborn et al., 2022; Danese et al., 2024; D'Haens G. R. et al., 2021; Louis et al., 2023; Sandborn et al., 2020; D'Haens et al., 2023; Magro et al., 2023b; Kobayashi et al., 2024; Peyrin-Biroulet et al., 2023), and the relevant flow chart was shown in Figure 1.

**FIGURE 2:**
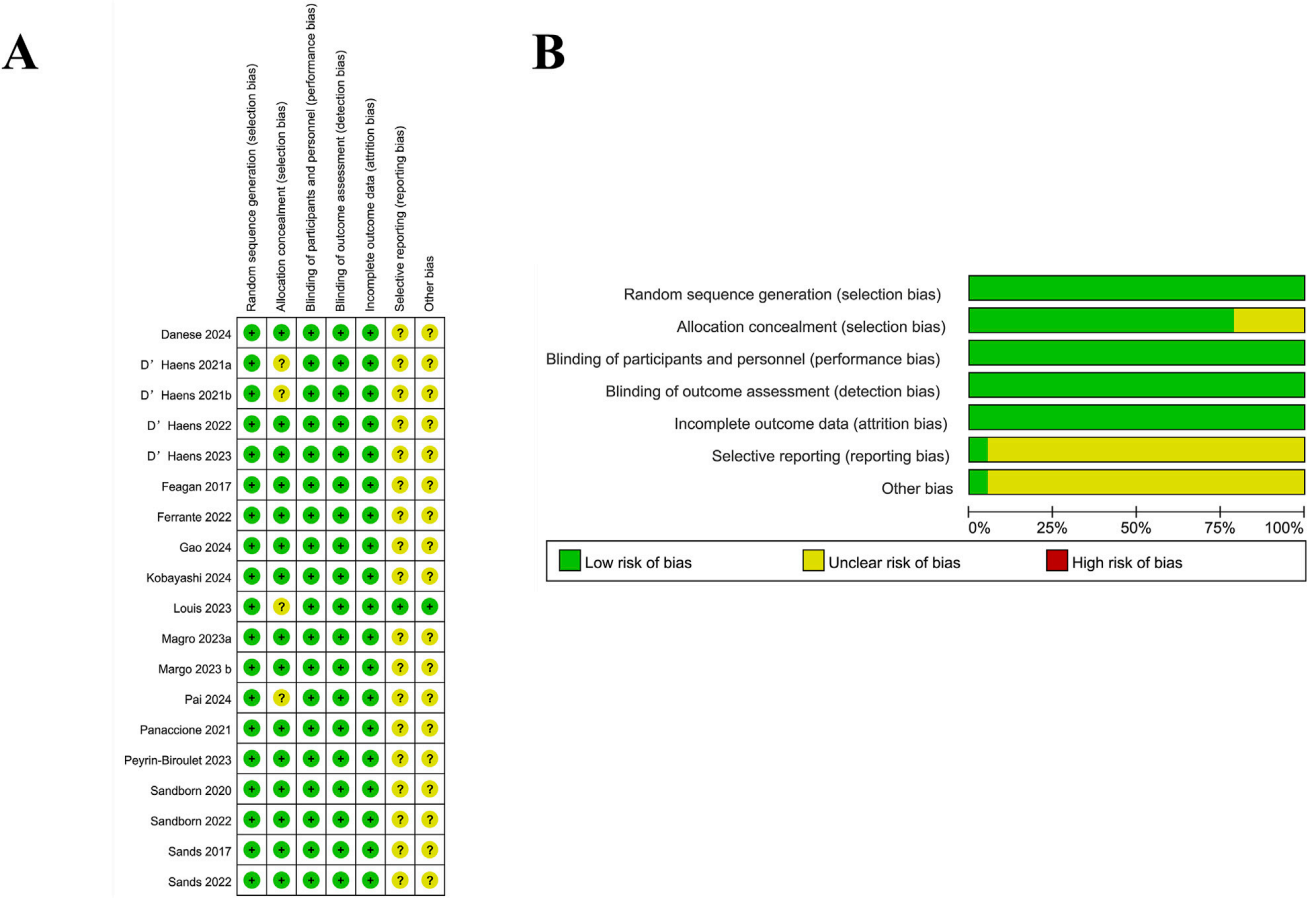
Risk of bias assessment for included studies. **(A)** Summary of the risk of bias for each included study across different domains. Green (+) indicates a low risk of bias, yellow (?) represents an unclear risk, and red (−) denotes a high risk of bias. **(B)** Proportion of studies with low, unclear, or high risk of bias in each domain. Most studies had a low risk of bias, with some uncertainty in selective reporting and other bias categories.

The quality of the included studies was analyzed by the RoB 2, which followed the guidelines outlined in the Cochrane Handbook for Systematic Reviews of Interventions. Additionally, the evaluation of the risk of bias was conducted by RevMan, and a total of 19 publications were finally included, including 25 RCTs. It was illustrated in Figures 2A,B that most studies exhibited the risk of bias with a low level within the random sequence generation, blinding of participants and personnel, outcome assessment, and completeness of outcome data. However, certain studies showed an unclear risk of bias regarding allocation concealment, selective reporting, and other potential sources of bias. Additionally, although the overall methodological quality was robust, it was difficult for some biases to be entirely ruled out. A total of 19 publications reporting data from 25 RCTs were incorporated in the systematic review and meta-analysis, among which 15 trials focused on CD, while 10 evaluated patients with UC. The detailed information was presented in Supplementary Table S1, the baseline patient characteristics were listed in Table 1, and the primary efficacy outcomes were shown in Supplementary Tables S4,S5.

**Table 1:** Baseline study characteristics of placebo-controlled double-blinded randomized trials of IL-23 p19 inhibitors in inflammatory bowel disease.

Study reference	Type of disease	Trial phase	Therapy period and time to primary outcome	Treatment Arms (n)	Female, n (%)	Mean Age (years, SD)	Baseline corticosteroid use, n (%)	Baseline immunomodulator use, n (%)	Ustekinumab failure history, n (%)	Vedolizumab failure history, n (%)
1. D’Haens 2022 (NCT03105128)	CD	III	Induction 12 weeks	Risankizumab 600 mg IV (336) Risankizumab 1200 mg IV (339) Placebo (175)	147 (44%) 156 (46%) 87 (50%)	38.3 (13.3) 37.0 (13.2) 37.1 (13.4)	102 (30%) 101 (30%) 50 (29%)	88 (26%) 73 (22%) 42 (24%)	43 (12.8%) 48 (14.2%) 19 (10.9%)	NR
2. D’Haens 2022 (NCT03104413)	CD	III	Induction 12 weeks	Risankizumab 600 mg IV (191) Risankizumab 1200 mg IV (191) Placebo (187)	99 (52%) 89 (47%) 88 (47%)	40.2 (13.6) 39.3 (12.9) 39.3 (13.5)	65 (34%) 62 (32%) 68 (36%)	36 (19%) 53 (28%) 40 (21%)	36 (18.8%) 33 (17.3%) 40 (21.4%)	NR
3. Ferrante 2022 (NCT03105102)	CD	III	Maintenance 52 weeks	Risankizumab 600 mg SC (157) Risankizumab 1200 mg SC (141) Placebo (164)	89 (57%) 60 (43%) 75 (46%)	39.1 (14.8) 37.0 (12.8) 38.0 (13.0)	51 (32%) 42 (30%) 51 (31%)	41 (26%) 40 (28%) 40 (24%)	18 (11.5%) 17 (12.1%) 15 (9.1%)	NR
4. Feagan 2017 (NCT02031276)	CD	II	Induction 12 weeks	Risankizumab 200 mg IV (41) Risankizumab 600 mg IV (41) Placebo (39)	26 (63%) 25 (61%) 23 (59%)	39.0 (13.0) 40.0 (13.0) 36.0 (14.0)	7 (17%) 9 (22%) 6 (15%)	7 (17%) 5 (12%) 8 (21%)	NR	NR
5. Gao 2024 (NCT03105128) (NCT03104413)	CD	III	Induction and Maintenance 12/52 weeks	Risankizumab 600 mg IV (70) Risankizumab 1200 mg IV (84) Placebo (44)	20 (28.6%) 27 (32.1%) 20 (45.5%)	36.9 (11.8) 34.1 (11.2) 34.8 (14.0)	17 (24.3) 15 (17.9) 9 (20.5)	32 (45.7%) 32 (38.1%) 17 (38.6%)	7 (14.9%) 7 (11.3%) 4 (11.1%)	5 (10.6) 4 (6.5) 5 (13.9)
6. D’Haens 2021 (NCT03105128)	CD	III	Induction 12 weeks	Risankizumab 600 mg IV (336) Risankizumab 1200 mg IV (339) Placebo (175)	NR	NR	NR	NR	NR	NR
7. Panaccione 2021 (NCT03104413)	CD	III	Induction 12 weeks	Risankizumab 600 mg IV (191) Risankizumab 1200 mg IV (191) Placebo (187)	NR	NR	NR	NR	NR	NR
8. Sands 2022 (NCT02891226)	CD	II	Induction 12 weeks	Mirikizumab 200 mg IV (31) Mirikizumab 600 mg IV (32) Mirikizumab 1000 mg IV (64) Placebo (64)	14 (45.2%) 18 (56.3%) 30 (46.9%) 36 (56.3%)	38.1 (11.8) 40.4 (13.3) 37.7 (13.1) 39.0 (13.0)	14 (45.2%) 7 (21.9%) 15 (23.4%) 21 (32.8%)	12 (38.7%) 10 (31.3%) 21 (32.8%) 19 (29.7%)	NR	5 (16.1%) 5 (15.6%) 6 (9.4%) 14 (21.9%)
9. Magro 2023 (NCT02891226)	CD	II	Induction 12 weeks	Mirikizumab 200 mg IV (65) Mirikizumab 600 mg IV (63) Mirikizumab 1000 mg IV (114) Placebo (120)	NR	NR	NR	NR	NR	NR
10. Pai 2024 (NCT02891226)	CD	II	Induction 12 weeks	Mirikizumab 200 mg IV (65) Mirikizumab 600 mg IV (63) Mirikizumab 1000 mg IV (115) Placebo (118)	NR	NR	NR	NR	NR	NR
11. Sands 2017 (NCT01714726)	CD	IIa	Induction 12 weeks	MEDI2070 700 mg IV (59) Placebo (60)	37 (62.7%) 37 (61.7%)	34.9 (11.2) 38.1 (10.7)	24 (40.7%) 24 (40.0%)	18 (30.5%) 14 (23.3%)	NR	NR
12. Sandborn 2022 (NCT03466411)	CD	II	Induction 12 weeks	Guselkumab 200 mg IV (61) Guselkumab 600 mg IV (63) Guselkumab 1200 mg IV (61) Placebo (61)	23 (37.7%) 27 (42.9%) 30 (49.2%) 24 (39.3%)	40.3 (13.67) 39.0 (14.35) 39.6 (13.72) 38.9 (12.95)	24 (39.3%) 19 (30.2%) 20 (32.8%) 24 (39.3%)	15 (24.6%) 18 (28.6%) 25 (41.0%) 26 (42.6%)	NR	6 (9.8%) 8 (12.7%) 3 (4.9%) 5 (8.2%)
13. Danese 2024 (NCT03466411)	CD	II	Maintenance 48 weeks	Guselkumab 200→100 mg IV→SC (61) Guselkumab 600→200 mg IV→SC (63) Guselkumab 1200→200 mg IV→SC (61) Placebo (61)	23 (38%) 27 (43%) 30 (49%) 24 (39%)	39.0 (29.0–49.0) 37.0 (26.0–50.0) 35.0 (30.0–51.0) 36.0 (29.0–47.0)	24 (39%) 19 (30%) 20 (33%) 24 (39%)	15 (25%) 18 (29%) 25 (41%) 26 (43%)	NR	6 (10%) 8 (13%) 3 (5%) 5 (8%)
14. D’Haens 2021	CD	II	Induction 12 weeks	Guselkumab 200 mg IV (50) Guselkumab 600 mg IV (50) Guselkumab 1200 mg IV (50) Placebo (51)	NR	NR	NR	NR	NR	NR
15. Louis 2023 (NCT03398148)	UC	III	Induction 12 weeks	Risankizumab 1200 mg IV (650) Placebo (325)	NR	NR	NR	NR	NR	NR
16. Sandborn 2020 (NCT02589665)	UC	II	Induction and Maintenance 12/40 weeks	Mirikizumab 50 mg IV (63) Mirikizumab 200 mg IV (62) Mirikizumab 600 mg IV (61) Placebo (63)	25 (39.7%) 25 (40.3%) 23 (37.7%) 27 (42.9%)	41.8 (14.1) 43.4 (14.7) 42.4 (13.4) 42.6 (13.5)	29 (46.0%) 25 (40.3%) 34 (55.7%) 33 (52.4%)	NR	NR	NR
17. D’Haens 2023 (NCT03518086) (NCT03524092)	UC	III	Induction and Maintenance 12/40 weeks	Mirikizumab 300 mg IV (868) Placebo (294)	338 (38.9%) 129 (43.9%)	42.9 (13.9) 41.3 (13.8)	351 (40.4%) 113 (38.4%)	211 (24.3%) 69 (23.5%)	NR	NR
18. Magro 2023 (NCT03518086) (NCT03524092)	UC	III	Induction and Maintenance 12/40 weeks	Mirikizumab 300 mg IV (868) Placebo (294)	NR	NR	NR	NR	NR	NR
19. Kobayashi 2024 (NCT03518086) (NCT03524092)	UC	III	Induction and Maintenance 12/40 weeks	Mirikizumab 300 mg IV (102) Placebo (35)	34 (33.3%) 12 (35.3%)	44.1 (14.0) 41.1 (13.5)	26 (25.5%) 9 (25.7%)	37 (36.3%) 15 (42.9%)	NR	10 (9.8%) 4 (11.4%)
20. Peyrin-Biroulet 2023 (NCT04033445)	UC	IIb	Induction 12 weeks	Guselkumab 200 mg IV (101) Guselkumab 400 mg IV (107) Placebo (105)	41 (40.6%) 48 (44.9%) 39 (37.1%)	43.3 (14.28) 40.4 (13.84) 41.2 (15.05)	41 (40.6%) 44 (41.1%) 40 (38.1%)	25 (24.8%) 27 (25.2%) 17 (16.2%)	NR	22 (21.8%) 22 (20.6%) 29 (27.6%)

IV: Intravenous; SC: Subcutaneous; NR, not reported.

### 3.2 Efficacy of IL-23 p19 inhibitors as induction therapy: Crohn’s disease

#### 3.2.1 Clinical remission and response

**FIGURE 3:**
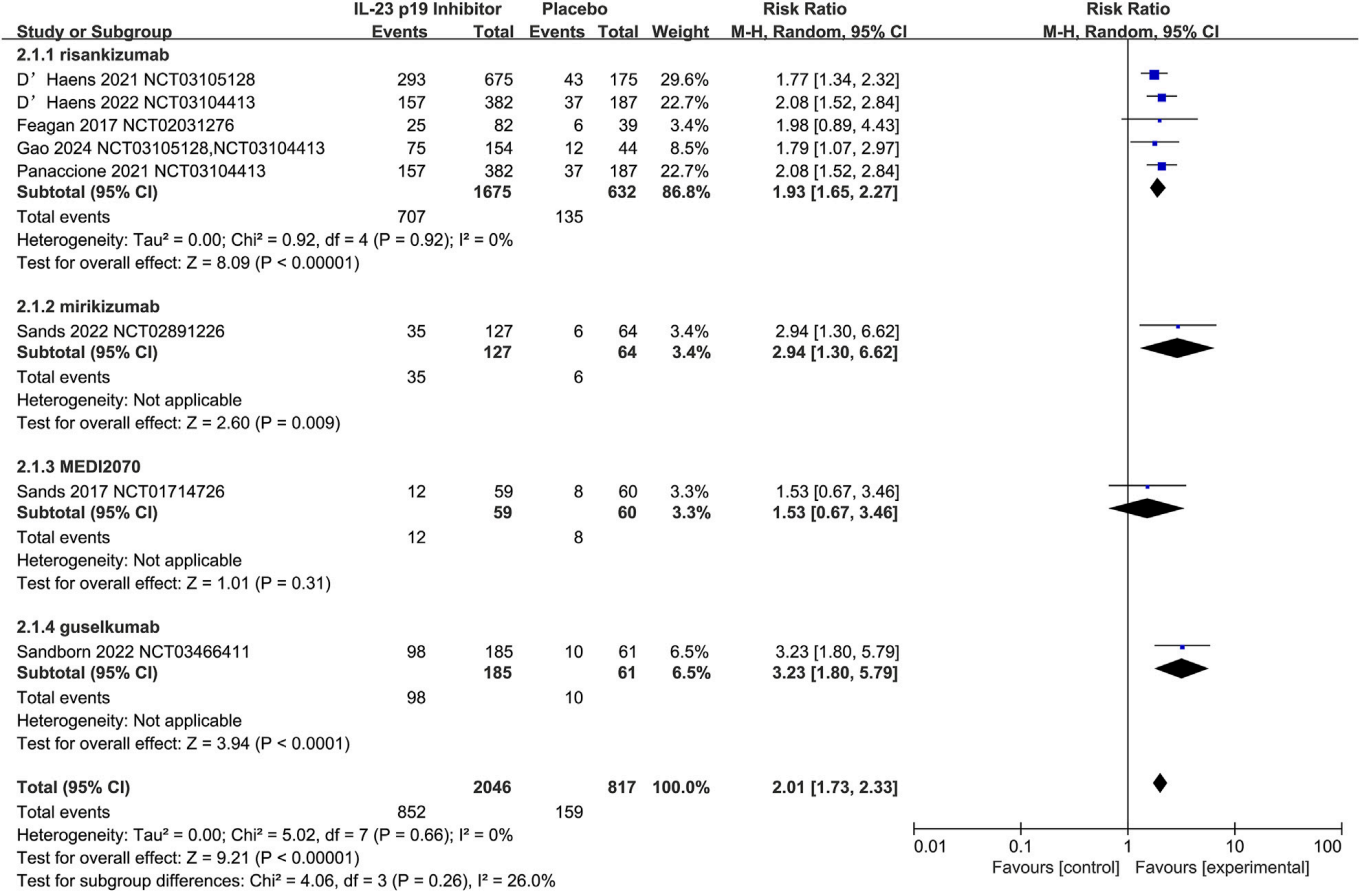
Efficacy of IL-23 p19 inhibitors in clinical remission in Crohn’s disease during the induction phase of therapy. Compared to placebo, IL-23 p19 inhibitor treatment significantly increased the induction of clinical remission (42.1% [1145/2721] vs. 20.4% [202/992], respectively; RR = 2.01, 95% CI 1.73–2.33). Heterogeneity among studies was low (I^2^ = 0%, p = 0.66).

The efficacy of IL-23 p19 inhibitors to serve as induction therapy (12 weeks) for CD was assessed by Nine studies. Compared with the placebo, IL-23 p19 inhibitors could significantly increase the induction of clinical remission (42.1% [1145/2721] vs. 20.4% [202/992], respectively; RR = 2.01, 95% CI 1.71–2.23; I^2^ = 0%, p = 0.68; Figure 3).

The risankizumab at doses of 600 mg (n = 515) and 1,200 mg (n = 475) was evaluated by eleven trials, and the superior efficacy over placebo in the clinical remission cod be demonstrated by both doses, with RR = 2.00 (95% CI 1.69–2.37) for 600 mg and RR = 1.87 (95% CI 1.57–2.22) for 1,200 mg. Additionally, the efficacy of risankizumab could be confirmed by the overall pooled estimate (RR = 1.94, 95% CI 1.72–2.19), without the observation of significant heterogeneity (I^2^ = 0%) (Supplementary Figure S1). Notably, the increase of the dose to 1,200 mg did not exhibit additional benefits, and the RR was slightly lower than that of 600 mg, which could suggest the saturation effect of the dose. Additionally, it was indicated that 600 mg might be the optimal dose, which could realize the balance between the efficacy and potential adverse effects.

Due to the limited sample sizes within this research, certain subgroups were excluded from the dose-response analysis risankizumab, mirikizumab, and guselkumab. Notably, the risankizumab 200 mg group and multi-dose trials of mirikizumab and guselkumab exhibited a lack of sufficient statistical power to detect significant dose-dependent effects, and it was necessary for future studies to further investigate the long-term impact of different dosing regimens on clinical remission and safety, to optimize the therapeutic strategies.

Additionally, the induction of clinical response was assessed by six studies, and it was found that 58.7% (887/1510) of the patients with IL-23 p19 inhibitors exhibited a clinical response, and the value was 30.0% (175/586) in the placebo group (RR = 1.88, 95% CI 1.62–2.17; I^2^ = 9%, p = 0.36; Supplementary Figure S1).

Regarding the CD, risankizumab at doses of 600 mg and 1,200 mg was significantly superior to the placebo in the aspects of clinical remission, with RR = 2.00 (95% CI 1.58–2.52) for 600 mg and RR = 2.03 (95% CI 1.58–2.60) for 1,200 mg (both p < 0.0001). Additionally, the efficacy of risankizumab could be confirmed by the overall pooled estimate (RR = 2.01, 95% CI 1.70–2.38), without the observation of significant heterogeneity (I^2^ = 0%) (Supplementary Figure S1). Nevertheless, there was no clear dose-dependent relationship observed during the research, and the 200 mg subgroup was excluded due to the small sample size, which limited the statistical power.

The dose-ranging trials for mirikizumab, MEDI2070, and guselkumab during the induction phase of CD treatment appeared to be limited, and further studies were necessary to clarify their dose-response relationships.

#### 3.2.2 Endoscopic response and remission

In a pooled analysis of nine studies, 35.0% (982/2812) of patients with IL-23 p19 inhibitors achieved an endoscopic response, and the value was 11.5% (113/983) in the placebo group (RR 2.98, 95% CI 2.49–3.58; I^2^ = 0%, p = 1.00; Supplementary Figure S1). In six of the studies, endoscopic remission also exhibited significant effects (20.8% [326/1570] vs. 5.4% [30/560], respectively; RR 3.48, 95% CI 2.43–4.98; I^2^ = 0%, p = 0.59; Supplementary Figure S1), and the results of the endoscopic studies were relatively consistent with high quality. Among the IL-23 p19 inhibitors, mirikizumab and guselkumab demonstrated greater efficacy in endoscopic response and remission compared with risankizumab. However, further verification was necessary due to the limited clinical studies on these two drugs.

#### 3.2.3 Histological response and remission

The histological data was generated in only two randomized controlled trials, and only two trials were available on histological response, which also exhibited significant efficacy for mirikizumab (58.4% [283/485] vs. 31.9% [76/238], respectively; RR 1.83, 95% CI 1.50–2.23; I^2^ = 0%, p = 0.95; Supplementary Figure S1). In terms of histological remission, mirikizumab also exhibited better results than the placebo (27.8% [135/485] vs. 10.5% [25/238], respectively; RR 2.65, 95% CI 1.78–3.94; I^2^ = 0%, p = 0.86; Supplementary Figure S1). However, although the results appeared to be consistent across studies, the data were relatively sparse with fair quality. Regarding the MEDI2070, risankizumab, and guselkumab, the data on histological outcomes were relatively lacking.

#### 3.2.4 SF/APS clinical response and remission

SF/APS were considered important indicators in the assessment of Crohn’s disease activity, and the effectiveness of treatment could be revealed by these changes. The data of SF/APS was obtained from seven randomized controlled trials, which showed better efficacy than placebo in terms of clinical response (62.5% [740/1184] vs. 39.7% [169/426], respectively; RR 1.56, 95% CI 1.38–1.77; I^2^ = 0%, p = 0.78; Supplementary Figure S1) and remission (40.8% [1053/2580] vs. 19.5% [174/893], respectively; RR 2.01, 95% CI 1.75–2.32; I^2^ = 0%, p = 0.90; Supplementary Figure S1). The drugs involved in the study were composed of risankizumab, mirikizumab, and guselkumab. However, further research was in urgent need due to the small amount of data and the evidence with moderate quality.

### 3.3 Efficacy of IL-23 p19 inhibitors as induction therapy: ulcerative colitis

#### 3.3.1 Clinical remission and response

**FIGURE 4:**
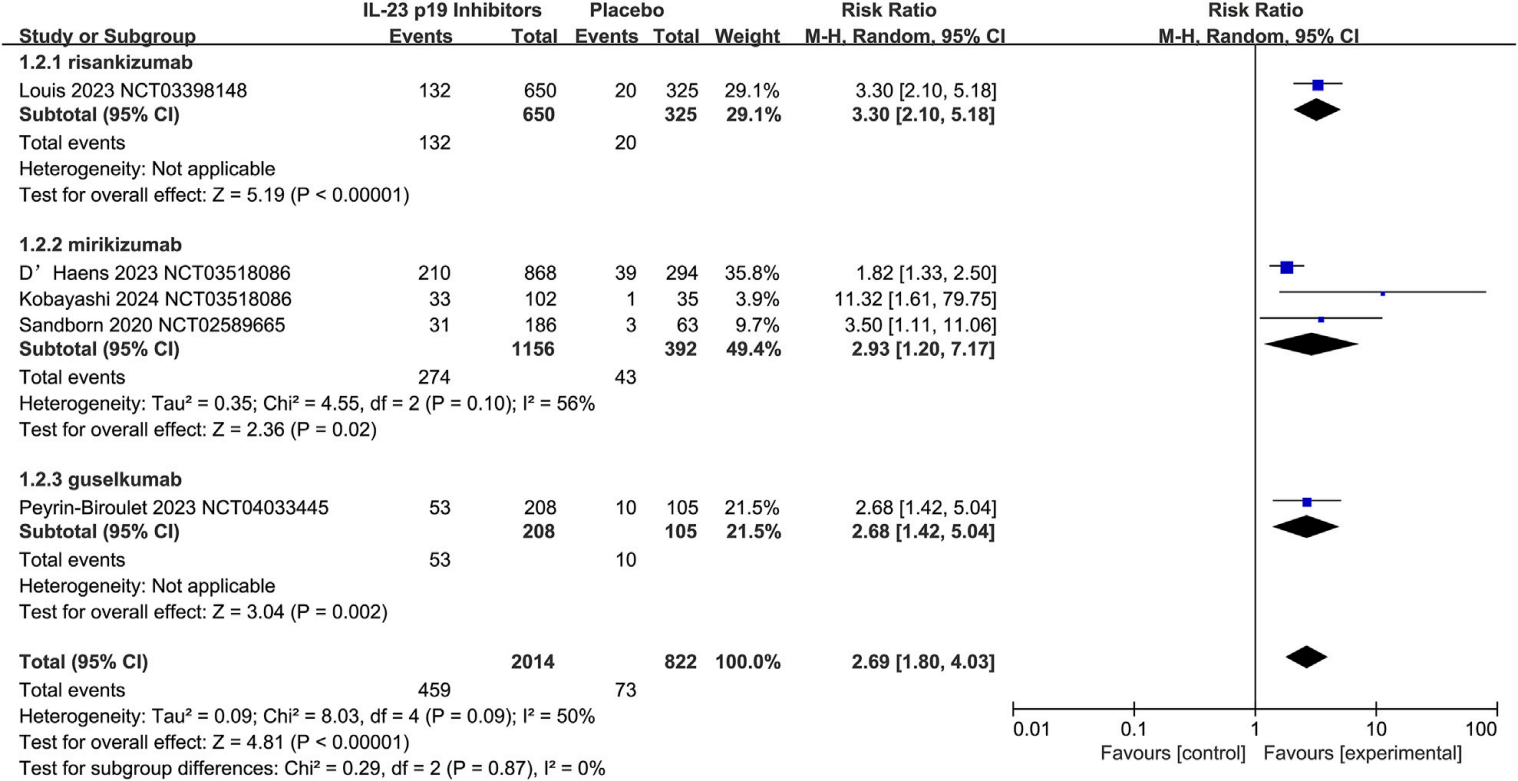
Efficacy of IL-23 p19 inhibitors in clinical remission in ulcerative colitis during the induction phase of therapy. Compared to placebo, IL-23 p19 inhibitor treatment significantly improved overall clinical remission, with 22.8% (459/2014) of patients achieving remission versus 8.9% (73/822) in the placebo group (RR = 2.69, 95% CI 1.80–4.03). Moderate heterogeneity was observed (I^2^ = 50%, p = 0.09), and the results were relatively consistent across subgroups.

The efficacy of IL-23 p19 inhibitors to serve as 12-week induction therapy for UC was evaluated by five studies, and the overall CR rate appeared to be relatively higher in the treatment group (22.8% [459/2014]) than in the placebo group (8.9% [73/822]; RR = 2.69, 95% CI 1.80–4.03; I^2^ = 50%, p = 0.09; Figure 4), and the results were relatively consistent across subgroups. Additionally, moderate heterogeneity could be observed in the study (I^2^ = 50%, p = 0.09), which was primarily driven by variations in the Mirikizumab subgroup (I^2^ = 56%) and differences among individual studies. Notably, an exceptionally high RR was reported by Kobayashi et al. (2024) (11.21, 95% CI 1.61–79.75), which substantially contributed to the overall heterogeneity. Despite these variations, there were no significant differences detected in treatment effects among IL-23 p19 inhibitors (p = 0.87).

Overall, the efficacy of IL-23 p19 inhibitors in terms of clinical response in UC could be further confirmed by these five studies, with response rates of 62.7% (1262/2014) in the treatment group and 35.3% (290/822) in the placebo group (RR = 1.92, 95% CI 1.56–2.35; I^2^ = 65%, p = 0.02; Supplementary Figure S2). It was indicated by the subgroup analysis that the risankizumab and guselkumab demonstrated greater efficacy than mirikizumab. However, the number of studies was relatively limited, no data were available on MEDI2070 (brazikumab) for UC induction therapy. The overall heterogeneity in clinical response analysis was moderate to high (I^2^ = 65%, p = 0.02), which was mainly driven by variations in the Mirikizumab subgroup (I^2^ = 75%). Notably, a markedly higher RR (3.13, 95% CI 1.68–5.83) was reported by Kobayashi et al. (2024), which could significantly contribute to the heterogeneity. Additionally, the confidence intervals might be widened by the smaller sample sizes in certain studies (e.g., Kobayashi et al., 2024; Sandborn et al., 2020), alongside the increased variability. Despite these inconsistencies, no significant differences were detected among IL-23 p19 inhibitors (p = 0.49), suggesting that the heterogeneity was primarily driven by individual variations rather than the inherent differences between agents.

#### 3.3.2 Endoscopic response and remission

The positive therapeutic effects of IL-23 p19 inhibitors on UC could also be indicated by the under endoscopy, and three studies have shown the outcome of endoscopic response, which were supported by substantial evidence (32.9% [343/1044] vs. 11.4% [56/493], respectively; RR 2.94, 95% CI 2.26–3.81; I^2^ = 0%, p = 0.76; Supplementary Figure S2). Additionally, the endoscopic remission outcomes were documented in five studies, which exhibited minimal heterogeneity and indicated the beneficial impact of IL-23 p19 inhibitors (23.0% [464/2014] vs. 10.2% [84/822], respectively; RR 2.31, 95% CI 1.57–3.40; I^2^ = 37%, p = 0.18; Supplementary Figure S2). Besides, low-to-moderate heterogeneity (I^2^ = 37%, p = 0.18) was revealed in the analysis of endoscopic remission in UC induction therapy, which could suggest the general consistency across the studies. Moreover, the Mirikizumab subgroup exhibited slight variability (I^2^ = 38%), which might be influenced by one study (Kobayashi et al., 2024) that reported a relatively higher RR (4.80, 95% CI 1.59–14.53), and the overall effect was estimated to remain stable. Additionally, no significant differences were observed between IL-23 p19 inhibitors (p = 0.73), which could reinforce the robustness of the findings.

#### 3.3.3 Histological response and remission

The evidence for the histologic efficacy of IL-23 p19 inhibitors in UC could be provided by five randomized controlled trials. Additionally, the effectiveness of the IL-23 p19 inhibitor in the treatment of this disease could be confirmed by both histologic response (26.1% [477/1828] vs. 12.8% [97/759], respectively; RR 2.05, 95% CI 1.06–3.97 I^2^ = 88%, p < 0.0001; Supplementary Figure S2) and histologic remission (11.8% [99/836] vs. 3.4% [13/388], respectively; RR 3.85, 95% CI 0.61 to 24.35; I^2^ = 83%, p = 0.01; Supplementary Figure S2). However, the evidence was relatively insufficient and required further confirmation. Besides, high heterogeneity (I^2^ = 88% and I^2^ = 83%, respectively) was demonstrated by the analysis of histologic response and remission in UC, which could indicate the substantial variability among the included studies. The heterogeneity was primarily driven by differences in drug efficacy, particularly in the Mirikizumab subgroup (I^2^ = 56%) and the Guselkumab subgroup, and no significant improvement could be observed in these groups. Additionally, the wide confidence interval in histologic remission (RR = 3.85, 95% CI 0.61–24.35) suggested a limited sample size and statistical instability.

### 3.4 Efficacy of IL-23 p19 inhibitors as maintenance therapy: Crohn’s disease

The maintenance outcomes (52 weeks) were assessed in only three CD trials, and only the outcomes of clinical remission and endoscopic response were available for meta-analysis.

**FIGURE 5:**
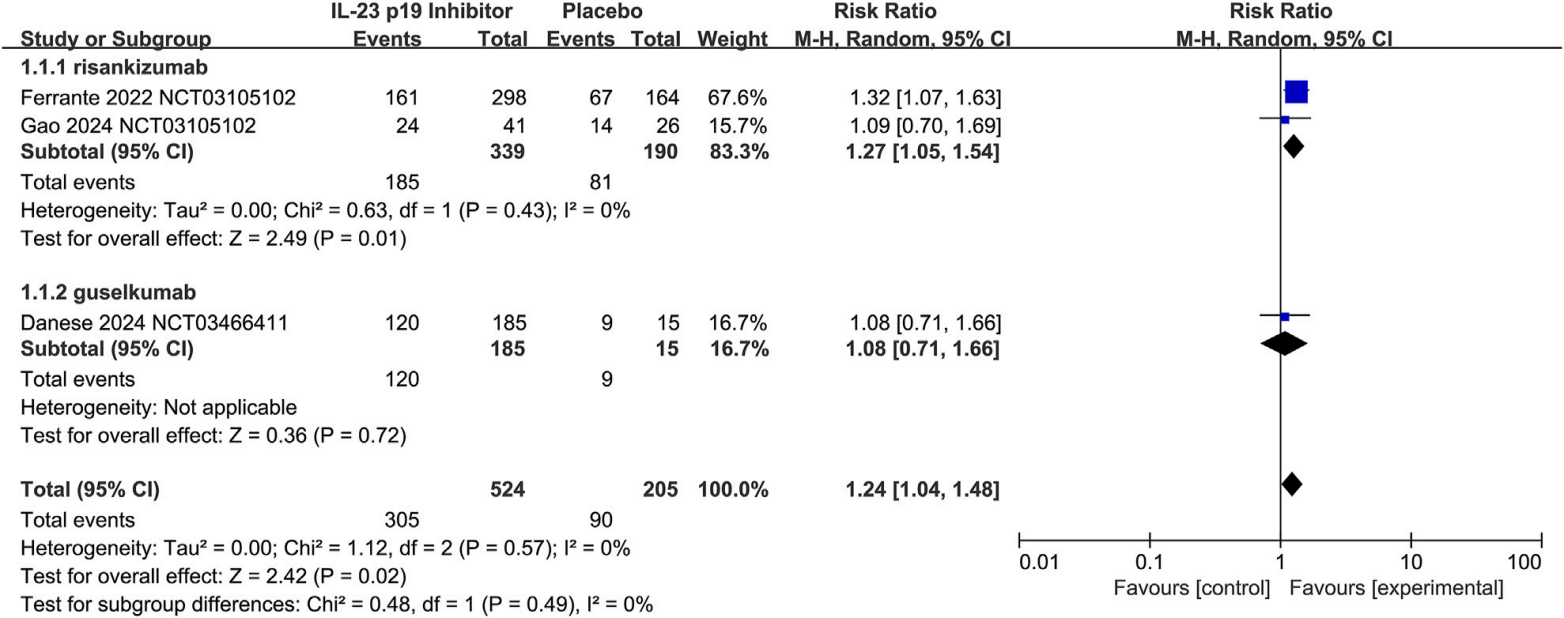
Efficacy of IL-23 p19 inhibitors in clinical remission of Crohn’s disease during the maintenance treatment phase. Compared to placebo, IL-23 p19 inhibitor therapy significantly improved the maintenance of clinical remission (58.2% [305/524] vs. 43.9% [90/205], respectively; RR = 1.24, 95% CI 1.04–1.48). Heterogeneity among studies was low (I^2^ = 0%, p = 0.57).

Additionally, therapies with IL-23 p19 inhibitors showed superior efficacy over placebo in the aspects of maintaining clinical remission (58.2% [305/524] vs. 43.9% [90/205], respectively; RR 1.24, 95% CI 1.04–1.48; I^2^ = 0%, p = 0.57; Figure 5), and this result was consistent in both two agents.

It could be revealed by the pooled analysis that IL-23 p19 inhibitors therapy was more effective than placebo in the preservation of endoscopic response (46.2% [242/524] vs. 22.9% [47/205], respectively; RR 1.95, 95% CI 1.37–2.79; I^2^ = 19%, p = 0.29; Supplementary Figure S3). Additionally, low heterogeneity (I^2^ = 19%, p = 0.29) was demonstrated in the analysis of endoscopic response maintenance in UC, which suggested the general consistency across the studies. Additionally, the Risankizumab subgroup showed some variability (I^2^ = 43%), which could be attributed to the differences in effect size between Ferrante et al. (2022) and Gao et al. (2024), and the overall heterogeneity remained at a low level. Additionally, no significant differences were observed between IL-23 p19 inhibitors (p = 0.39), which could reinforce the robustness of the findings.

### 3.5 Efficacy of IL-23 p19 inhibitors as maintenance therapy: ulcerative colitis

The maintenance (40 weeks) outcomes were evaluated in only four UC trials, and the data on clinical remission and endoscopic response was available for meta-analysis. Notably, some data gaps remained in this research, and a comprehensive meta-analysis was required to be further performed.

**FIGURE 6:**
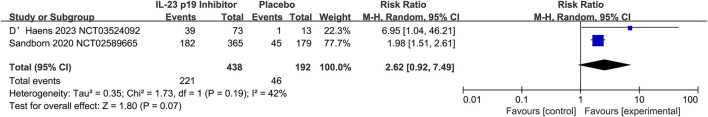
Efficacy of IL-23 p19 inhibitors in clinical remission of ulcerative colitis during the maintenance treatment phase. Compared to placebo, only two studies have demonstrated the efficacy of IL-23 p19 inhibitors in maintaining clinical remission in UC (50.5% [221/438] vs. 24.0% [46/192], respectively; RR = 2.62, 95% CI 0.92–7.49). Moderate heterogeneity was observed among studies (I^2^ = 42%, p = 0.19).

Only two studies have shown the function of IL-23 p19 inhibitors in maintaining clinical remission in UC (50.5% [221/438] vs. 24.0% [46/192], respectively; RR 2.62, 95% CI 0.92–7.49; I^2^ = 42%, p = 0.19; Figure 6). Additionally, the moderate heterogeneity (I^2^ = 42%, p = 0.19) could be demonstrated by the analysis of clinical remission maintenance in UC, and it could indicate the differences among the included studies. Additionally, the heterogeneity was primarily driven by D’Haens 2023, which reported a much higher RR (6.95, 95% CI 1.04–46.21) versus the Sandborn et al. (2020) (RR = 1.98, 95% CI 0.51–2.61). Additionally, it could be suggested by the wide confidence interval (RR = 2.62, 95% CI 0.92–7.49) that the statistical stability was relatively limited, which might be attributed to the small sample size.

Regarding the UC maintenance trials, data from four studies revealed that IL-23 p19 inhibitor therapy was superior to placebo in terms of endoscopic response (54.6% [475/870] vs. 28.3% [112/396], respectively; RR 2.03, 95% CI 1.71–2.39; I^2^ = 0%, p = 0.99; Supplementary Figure S4). In terms of the pooled analysis, this superior efficacy could be consistently demonstrated by all three drugs.

### 3.6 Safety outcomes

#### 3.6.1 Induction therapy

The safety comparison between IL-23 p19 inhibitors and placebo was outlined in Table 2. According to induction trial results, the treatment with IL-23 p19 inhibitors for IBD patients did not result in a substantially increasing trend in the overall risk of adverse events (AE) compared to the placebo administration (CD: RR 0.88, 95% CI 0.82–0.94; I^2^ = 2%, p = 0.41; UC: RR 0.92, 95% CI 0.82–1.03; I^2^ = 0%, p = 0.54; Supplementary Figure S5), but the data showed no statistical significance in UC patients. Additionally, IL-23 p19 inhibitors have been confirmed to exhibit effects on the decrease of the incidence of SAEs during IBD induction therapy in both CD and UC patients (CD: RR 0.46, 95% CI 0.35–0.60; I^2^ = 34%, p = 0.17; UC: RR 0.47, 95% CI 0.28–0.78; I^2^ = 0%, p = 0.74; Supplementary Figure S5). In addition, IL-23 p19 also exhibited the functions to significantly reduce the incidence of AE-related discontinuation events during the treatment of CD and UC (CD: RR 0.37, 95% CI 0.24–0.58; I^2^ = 8%, p = 0.37; UC: RR 0.20, 95% CI 0.11–0.35; I^2^ = 0%, p = 0.74; Supplementary Figure S5).

**Table 2:** Summary of safety outcomes.

Study reference	Type of disease	Therapy period and time to primary outcome	Treatment Arms (n)	AEs, n (%)	Serious AEs, n (%)	AE-related discontinuation, n (%)	Infections, n (%)	Serious infections, n (%)
1. D’Haens 2022 (NCT03105128)	CD	Induction 12 weeks	Risankizumab 600 mg IV (373) Risankizumab 1200 mg IV (372) Placebo (186)	210 (56%) 191 (51%) 105 (56%)	27 (7%) 14 (4%) 28 (15%)	9 (2%) 7 (2%) 14 (8%)	NR	3 (1%) 2 (1%) 7 (4%)
2. D’Haens 2022 (NCT03104413)	CD	Induction 12 weeks	Risankizumab 600 mg IV (206) Risankizumab 1200 mg IV (205) Placebo (207)	98 (48%) 121 (59%) 137 (66%)	10 (5%) 9 (4%) 26 (13%)	2 (1%) 5 (2%) 17 (8%)	NR	1 (<1%) 2 (1%) 5 (2%)
3. Ferrante 2022 (NCT03105102)	CD	Maintenance 52 weeks	Risankizumab 600 mg SC (179) Risankizumab 1200 mg SC (179) Placebo (184)	128 (72%) 129 (72%) 135 (73%)	12 (7%) 21 (12%) 23 (13%)	3 (2%) 6 (3%) 6 (3%)	NR	5 (3%) 8 (4%) 7 (4%)
4. Feagan 2017 (NCT02031276)	CD	Induction 12 weeks	Risankizumab 200 mg IV (41) Risankizumab 600 mg IV (41) Placebo (39)	32 (78%) 31 (76%) 32 (82%)	9 (22%) 9 (22%) 9 (22%)	5 (12%) 1 (2%) 6 (15%)	11 (27%) 13 (32%) 11 (28%)	NR
5. Gao 2024 (NCT03105128) (NCT03104413)	CD	Induction 12 weeks	Risankizumab 600 mg IV (70) Risankizumab 1200 mg IV (84) Placebo (44)	30 (42.9%) 43 (51.2%) 26 (59.1%)	4 (5.7%) 0 5 (11.4%)	2 (2.9%) 0 2 (4.5%)	NR	0 0 1 (2.3%)
6. Gao 2024 (NCT03105102)	CD	Maintenance 52 weeks	Risankizumab 180 mg IV (21) Risankizumab 360 mg IV (20) Placebo (26)	12 (57.1%) 13 (65.0%) 19 (73.1%)	1 (4.8%) 3 (15.0%) 5 (19.2%)	0 0 1 (3.8%)	NR	0 1 (5.0%) 1 (3.8%)
7. D’Haens 2021 (NCT03105128)	CD	Induction 12 weeks	Risankizumab 600 mg IV Risankizumab 1200 mg IV Placebo	NR	NR	NR	NR	NR
8. Panaccione 2021 (NCT03104413)	CD	Induction 12 weeks	Risankizumab 600 mg IV Risankizumab 1200 mg IV Placebo	NR	NR	NR	NR	NR
9. Sands 2022 (NCT02891226)	CD	Induction 12 weeks	Mirikizumab 200 mg IV (31) Mirikizumab 600 mg IV (32) Mirikizumab 1000 mg IV (64) Placebo (64)	18 (58.1%) 21 (65.6%) 42 (65.6%) 45 (70.3%)	0 3 (9.4%) 2 (3.1%) 7 (10.9%)	1 (3.2%) 3 (9.4%) 0 4 (6.3%)	NR	NR
10. Magro 2023 (NCT02891226)	CD	Induction 12 weeks	Mirikizumab 200 mg IV (65) Mirikizumab 600 mg IV (63) Mirikizumab 1000 mg IV (114) Placebo (120)	NR	NR	NR	NR	NR
11. Pai 2024 (NCT02891226)	CD	Induction 12 weeks	Mirikizumab 200 mg IV (65) Mirikizumab 600 mg IV (63) Mirikizumab 1000 mg IV (115) Placebo (118)	NR	NR	NR	NR	NR
12. Sands 2017 (NCT01714726)	CD	Induction 12 weeks	MEDI2070 700 mg IV (59) Placebo (60)	40 (67.8%) 41 (68.3%)	5 (8.5%) 5 (8.3%)	5 (8.5%) 6 (10.0%)	4 (6.8%) 11 (18.3%)	NR
13. Sandborn 2022 (NCT03466411)	CD	Induction 12 weeks	Guselkumab 200 mg IV (73) Guselkumab 600 mg IV (73) Guselkumab 1200 mg IV (73) Placebo (70)	32 (43.8%) 37 (50.7%) 31 (42.5%) 42 (60.0%)	3 (4.1%) 4 (5.5%) 1 (1.4%) 4 (5.7%)	1 (1.4%) 0 1 (1.4%) 2 (2.9%)	9 (12.3%) 13 (17.8%) 11 (15.1%) 15 (21.4%)	1 (1.4%) 2 (2.7%) 0 0
14. Danese 2024 (NCT03466411)	CD	Maintenance 48 weeks	Guselkumab 200→100 mg IV→SC (73) Guselkumab 600→200 mg IV→SC (73) Guselkumab 1200→200 mg IV→SC (73) Placebo (70)	52 (71%) 59 (81%) 51 (70%) 46 (66%)	6 (8%) 5 (7%) 5 (7%) 6 (9%)	5 (7%) 2 (3%) 6 (8%) 4 (6%)	25 (34%) 30 (41%) 25 (34%) 17 (24%)	2 (3%) 2 (3%) 1 (1%) 1 (1%)
15. D’Haens 2021	CD	Induction 12 weeks	Guselkumab 200 mg IV (50) Guselkumab 600 mg IV (50) Guselkumab 1200 mg IV (50) Placebo (51)	NR	NR	NR	NR	NR
16. Louis 2023 (NCT03398148)	UC	Induction 12 weeks	Risankizumab 1200 mg IV (650) Placebo (325)	NR	NR	NR	NR	NR
17. D’Haens 2023 (NCT03518086)	UC	Induction 12 weeks	Mirikizumab 300 mg IV (958) Placebo (321)	426 (44.5%) 148 (46.1%)	27 (2.8%) 17 (5.3%)	15 (1.6%) 23 (7.2%)	145 (15.1%) 45 (14.0%)	7 (0.7%) 2 (0.6%)
18. D’Haens 2023 (NCT03524092)	UC	Maintenance 40 weeks	Mirikizumab 200 mg SC (389) Placebo (192)	251 (64.5%) 132 (68.8%)	13 (3.3%) 15 (7.8%)	6 (1.5%) 16 (8.3%)	93 (23.9%) 44 (22.9%)	3 (0.8%) 3 (1.6%)
19. Magro 2023 (NCT03518086)	UC	Induction 12 weeks	Mirikizumab 300 mg IV (868) Placebo (294)	NR	NR	NR	NR	NR
20. Magro 2023 (NCT03524092)	UC	Maintenance 40 weeks	Mirikizumab 200 mg SC (365) Placebo (179)	NR	NR	NR	NR	NR
21. Kobayashi 2024 (NCT03518086)	UC	Induction 12 weeks	Mirikizumab 300 mg IV (102) Placebo (35)	48 (47.1%) 19 (54.3%)	3 (2.9%) 3 (8.6%)	2 (2.0%) 6 (17.1%)	18 (17.6%) 6 (17.1%)	1 (1.0) 1 (2.9)
22. Kobayashi 2024 (NCT03524092)	UC	Maintenance 40 weeks	Mirikizumab 200 mg SC (47) Placebo (25)	42 (89.4%) 22 (88.0%)	2 (4.3%) 2 (8.0%)	2 (4.3%) 3 (12.0%)	20 (42.6%) 11 (44.0%)	0 0
23. Peyrin-Biroulet 2023 (NCT04033445)	UC	Induction 12 weeks	Guselkumab 200 mg IV (101) Guselkumab 400 mg IV (107) Placebo (105)	45 (44.6%) 53 (49.5%) 59 (56.2%)	1 (1.0%) 3 (2.8%) 6 (5.7%)	1 (1.0%) 0 3 (2.9%)	14 (13.9%) 10 (9.3%) 13 (12.4%)	0 0 2 (1.9%)

IV: Intravenous; SC: Subcutaneous; NR, not reported.

Besides, the pooled analysis of induction studies showed that the infection risk in patients with CD and UC with IL-23 p19 inhibitors was not significantly different from that in placebo (CD: RR 0.74, 95% CI 0.46–1.18; I^2^ = 29%, p = 0.24; UC: RR 1.05, 95% CI 0.80–1.36; I^2^ = 0%, p = 0.92; Supplementary Figure S5), and no statistical significance was observed. However, the incidence of serious infection showed a decreasing trend (CD: RR 0.28, 95% CI 0.13–0.61; I^2^ = 0%, p = 0.39; UC: RR 0.56, 95% CI 0.18–1.69; I^2^ = 9%, p = 0.33; Supplementary Figure S5).

#### 3.6.2 Maintenance therapy

According to maintenance trial results, treatment of IBD patients with an IL-23 p19 inhibitor showed no connection with the significantly greater AEs compared with placebo (CD: RR 1.00, 95% CI 0.89–1.13; I^2^ = 29%, p = 0.25; UC: RR 0.96, 95% CI 0.87–1.06; I^2^ = 0%, p = 0.44; Supplementary Figure S6), but the results were not statistically significant. Additionally, it did not show a strong connection with serious adverse events versus the placebo (CD: RR 0.73, 95% CI 0.48–1.10; I^2^ = 0%, p = 0.80; UC: RR 0.44, 95% CI 0.22–0.86; I^2^ = 0%, p = 0.83; Supplementary Figure S6). In addition, the incidence of AE-related discontinuation events in CD and UC treatment showed no obvious difference with placebo (CD: RR 0.82, 95% CI 0.40–1.70; I^2^ = 0%, p = 0.64; UC: RR 0.21, 95% CI 0.09–0.48; I^2^ = 0%, p = 0.51; Supplementary Figure S6).

Regarding the maintenance treatment outcomes of UC patients, IL-23 p19 inhibitors exhibited no obvious effects on infection (RR 1.03, 95% CI 0.78–1.35; I^2^ = 0%, p = 0.81, Supplementary Figure S6), and the result was not statistically significant. When the trial data were combined with serious infection rates, the risk of these events was not significantly different with IL-23 p19 inhibitor therapy compared with placebo (CD: RR 0.99, 95% CI 0.45–2.19; I^2^ = 0%, p = 0.86; UC: RR 0.49, 95% CI 0.10–2.42; Supplementary Figure S6), and the results of this analysis showed no statistical significance.

## 4 Discussion

Despite the substantial progress of biologic therapies in the management of IBD, particularly in the decrease of surgical interventions, morbidity, and mortality, a considerable proportion of patients tended to fail to respond, lose therapeutic effects over time, or experience intolerance or adverse events (Queiroz and Regueiro, 2020). Moreover, many individuals were unable to achieve sustained remission or suffered from treatment-related complications, which significantly limited the long-term effectiveness and broad applicability of current biological agents. Besides, the urgent need for novel therapies with improved safety and efficacy was underscored by the above limitations.

IL-23 p19-targeted therapies were considered a promising option for patients who fail to respond or lose response to currently available biologics, such as anti-TNF agents. Additionally, they could offer renewed hope for individuals with refractory IBD, contributing to the advancement of personalized treatment strategies. Different from the broad-spectrum immunosuppressants, IL-23 p19 inhibitors could selectively target critical components of the inflammatory cascade without broadly suppressing the immune system (Bourgonje et al., 2024). As a result, a lower risk of immunosuppression-related complications could be obtained in this method, such as infections. Additionally, this action mechanism marked an important shift in the therapeutic approach for IBD.

Data from numerous studies on IL-23 p19 inhibitors for IBD was synthesized in this meta-analysis, revealing substantial clinical, endoscopic, and histological efficacy. Nevertheless, a noteworthy subset of patients continued to exhibit non-responsiveness, loss of therapeutic efficacy over time, alongside the intolerance or adverse effects associated with these therapies.

Among the reviewed studies, the pharmacological agents employed for IBD treatment were composed of risankizumab, mirikizumab, MEDI2070, and guselkumab. Notably, MEDI2070 was excluded from the analysis of UC treatment, and the number of research focused on UC was comparatively limited relative to CD.

Several key findings that might affect treatment decisions were yielded by the meta-analysis. First, the clinical response and remission of IL-23 p19 inhibitors were superior to the placebo, and it was indicated by the analysis based on dosimetry that high doses were connected with clinical efficacy. In addition, more studies were focused on CD rather than UC. Additionally, a certain effect in the treatment of UC patients could be indicated despite the absence of a separate meta-analysis. It was demonstrated by the endoscopic evaluations that the IL-23 p19 inhibitors exhibited robust efficacy. Notably, the endoscopic effectiveness in patients with UC was further corroborated within the scope of the included studies. Additionally, the above findings could further underscore the therapeutic potential of IL-23 p19 inhibitors in the aspects of clinical and histological improvements, alongside the realization of significant endoscopic healing, which could provide comprehensive therapeutic benefits for IBD patients. Additionally, the limit in histological data might be attributed to the pervasive inflammation and ulceration typical of IBD, which could complicate the technical challenges of obtaining and interpreting tissue specimens. Nevertheless, the efficacy of IL-23 p19 targeted therapy could be confirmed and emphasized by the current evidence. In addition, treatment with these medications did not demonstrate a higher incidence versus the placebo. Furthermore, it was indicated by the research that the p19 subunit of IL-23 exhibited selectivity by specifically inhibiting the IL-12-dependent T-cell pathways that were involved in infection and cancer development. Therefore, targeting IL-23 p19 was confirmed as a safer therapeutic approach. However, the assertions mentioned above remained theoretical, which underscored the further clinical trials for validation.

Additionally, this study was characterized by several limitations (Zhou et al., 2023): Although a rigorous search strategy was implemented, some certain literature might remain undiscovered, and the included studies were limited to published sources. Furthermore, some information was obtained from the conference abstracts, which restricted the operation of subgroup and sensitivity analyses (Kaplan and Windsor, 2021). Randomized trials focused on the application of IL-23 p19 antagonists for treating UC were relatively lacking, and the available data was featured by a small sample size (Lewis et al., 2023). The diversity in experimental drug types and dosages, as well as variations in the concurrent application of immunomodulators, corticosteroids, and other medications among patients, were lacking for a standardized criterion across the studies. Therefore, it was necessary for subsequent investigations to elucidate the personalized response mechanisms to biologics among diverse patient populations and innovate novel therapeutic approaches. Furthermore, it was indispensable to gain more attention to the conduction of rigorous randomized controlled trials of superior quality, which could contribute to the confirmation and broadening of our discoveries (Piovani et al., 2019). Some limitations in certain included studies were revealed by the assessment of the risk of bias, particularly regarding the aspects of allocation concealment and selective reporting, which exhibited effects on the reliability of the findings. Additionally, a detailed description of random sequence generation was lacking in some trials, which might introduce potential selection bias. Additionally, only histological or endoscopic outcomes were reported in certain studies, and comprehensive clinical response data were lacking, which could limit the overall evaluation of IL-23 p19 inhibitors resulting in selective reporting bias. Moreover, the short follow-up duration in some trials restricted the assessment of a long-term index. Future research should focus on the enhancement of transparency in randomization methods, ensurement of complete and standardized outcome reports, and extension of follow-up periods to improve the robustness and clinical applicability of the findings (Kotla and Rochev, 2023). Several subgroup analyses were restricted by small sample size, particularly for those groups that evaluated the different dosages of risankizumab, mirikizumab, and guselkumab. In particular, the 200 mg risankizumab group and the multi-dose trials for mirikizumab and guselkumab lacked sufficient statistical power in the detection of predominant dose-response relationships. Additionally, certain studies were composed of relatively few participants, which resulted in wider confidence intervals and reduced precision in effect estimates, such as Kobayashi et al. (2024) and Sandborn et al. (2020). Therefore, it was necessary for future research to pay more attention to larger, well-powered RCTs to confirm these findings and establish optimal dosing strategies (Cai et al., 2021). Moderate to high heterogeneity was observed in clinical remission (I^2^ = 50%) and response (I^2^ = 65%) analyses and it was mainly driven by variability in the mirikizumab subgroup and an outlier effect size reported in Kobayashi et al. (2024), which could indicate the differences within patient populations, disease severity, or study design rather than the drug efficacy. Additionally, there were no significant differences found between IL-23 p19 inhibitors, suggesting that the heterogeneity might be affected by the study-level factors rather than the inherent differences among agents (Luo et al., 2022). Additionally, endoscopic and histologic outcomes also exhibited inconsistency, although the endoscopic response and its maintenance were relatively consistent across studies, notable heterogeneity was observed in endoscopic remission (I^2^ = 42%) and especially in histologic response and remission (I^2^ = 88% and 83%, respectively). Besides, it might be attributed to the smaller studies with divergent methodologies and wide confidence intervals, which limited the confidence in these results (Cheifetz et al., 2021). Notably, it was demonstrated by the meta-analysis that IL-23 p19 inhibitors showed no connection with an increased risk of adverse events compared to placebo during either induction or maintenance phases. Additionally, the agents were found to significantly reduce the risk of serious adverse events and AE-related discontinuations during induction therapy in both Crohn’s disease and ulcerative colitis. Although the incidence of serious infections appeared to be lower in several subgroups, most of these findings exhibited no statistical significance, which was mainly attributed to the limited event rates and wide confidence intervals. Additionally, the values of overall AEs, serious AEs, infections, and discontinuations remained similar between IL-23 p19 inhibitors and placebo during the maintenance therapy. However, despite the encouraging results mentioned above, the current safety evidence was constrained by short follow-up durations and the small sample size in certain studies, which might limit the diagnosis of adverse events. Therefore, it was necessary for future research to include larger patient cohorts, longer follow-up periods, and head-to-head comparisons with other biologics, to obtain the long-term safety profile.

It was essential for future research to pay more attention to the long-term follow-up studies, in order to assess the sustained efficacy and potential delayed adverse effects of IL-23 p19 inhibitors in IBD. Additionally, stratified analyses across diverse patient subgroups, which were defined by age, sex, ethnicity, disease duration, and comorbidities, were warranted to identify populations with differential treatment responses. In addition, head-to-head comparisons with other biological agents were necessary to clarify their relative efficacy and safety profiles (Katsanos and Papadakis, 2017), such as IL-17 or TNF inhibitors.

Additionally, the therapeutic outcomes could be further optimized by the investigation of combination regimens with conventional immunosuppressants or other biologics. Besides, future trials should focus on a larger sample size and broader geographic representation, in order to enhance the generalizability and robustness of findings. Moreover, the integration of randomized controlled trials with real-world evidence (RWE) (Stern et al., 2022) and the incorporation of patient-reported outcomes (PROs) (Hammert and Calfee, 2020) would be essential in the evaluation of its effects on life quality, symptom control, and overall patient satisfaction.

Although the direct head-to-head trials were relatively lacking, it could be suggested by the indirect comparisons from network meta-analyses that IL-23 p19 inhibitors might exhibit comparable or superior efficacy to anti-TNF agents in biologic-naïve patients, alongside a more favorable safety profile in long-term application. Additionally, although further data was required, their efficacy in anti-TNF-experienced patients appeared to be more consistent than that of other newer biologics.

Given the favorable safety and immunologic selectivity, IL-23 p19 inhibitors might be optimal as the second-line options after anti-TNF failure. Additionally, it was revealed by emerging evidence that the potential benefits could be found in first-line application among moderate-to-severe cases (Lusetti et al., 2024), especially those at higher risk of infections or with comorbidities.

Besides, special populations such as elderly patients, those with prior malignancy, or those with heightened infection risk particularly benefited from IL-23 p19 inhibitors due to their targeted mechanisms and minimal systemic immunosuppression. However, the targeted studies were warranted due to the limited data.

Based on current evidence, the IL-23 p19 inhibitors could be considered in a stratified treatment model, which could serve as the first-line options for biologic-naïve patients with high infection risk or comorbidities, alongside the second-line agents for those with anti-TNF failure. Additionally, tailoring treatment based on patient characteristics, disease behavior, and prior biologic exposure was essential for the optimization of clinical outcomes.

Moreover, the additional considerations based on real-world conditions tended to be involved in the clinical decision-making process. Such as cost, availability, patient preference, and route of administration, which required more attention in the incorporation of IL-23 p19 inhibitors into the treatment strategies. Although further comparative data were relatively lacking, IL-23 p19 inhibitors could offer a more favorable benefit-risk profile versus other advanced therapies such as IL-12/23 inhibitors or Janus kinase (JAK) inhibitors. Finally, long-term real-world data and post-marketing studies were necessary for the validation of the sustained efficacy and safety of IL-23 p19 inhibitors, particularly for diverse and high-risk patient populations.

It was indicated by the findings of this study that IL-23 p19 antagonists could enhance both clinical remission and response rates in individuals with moderate to severe active IBD, which could demonstrate a favorable safety profile with minimal SAEs. Nonetheless, some limitations still existed in current trials, such as small sample size and short follow-up time. Consequently, it was suggested that future research should focus on the investigation of the clinical tolerability, endoscopic outcomes, precise efficacy, and long-term safety profile of these novel agents, which contributed to the development of these technologies into a pivotal area of study in the coming years.

## Data Availability

The original contributions presented in the study are included in the article/Supplementary Material, further inquiries can be directed to the corresponding author.
