# Supplementary material for: Efficacy and safety of IL-23 p19 inhibitors in the treatment for inflammatory bowel disease: a systematic review and meta-analysis
Source: Front Pharmacol. 2025 Apr 28;16:1490667. doi: 10.3389/fphar.2025.1490667 (PMC12066446; doi:10.3389/fphar.2025.1490667)
Supplement: Supplementary file 1 [file DataSheet1.docx]

Supplementary Material

# Supplementary Data

1.1 To ensure a comprehensive and systematic search, the following predefined search terms were developed based on MeSH terms, synonyms, and Boolean operators. These terms were used across multiple databases, including PubMed, Embase, and Cochrane Library.

(1) Inflammatory Bowel Disease (IBD) Related Terms

(a) MeSH Term: “Inflammatory Bowel Diseases”[Mesh]

(b) Free Text Search (Title/Abstract): "Inflammatory Bowel Disease" OR "IBD", "Crohn’s Disease" OR "Crohn Disease" OR "CD", "Ulcerative Colitis" OR "UC", "Regional Enteritis" OR "Ileocolitis" OR "Terminal Ileitis", "Granulomatous Colitis" OR "Idiopathic Proctocolitis"

(2) IL-23 p19 Inhibitors Related Terms

(a) General Mechanism Terms: "IL-23 p19" OR "Interleukin-23 p19" OR "IL-23 alpha subunit", "IL-23 blockade" OR "IL-23 inhibition", "Interleukin 23 antagonist" OR "Interleukin 23 inhibitor"

(b) Specific Drug Names:

Risankizumab: "Risankizumab" OR "Skyrizi" OR "BI 655066" OR "ABBV-066"

Mirikizumab: "Mirikizumab" OR "LY3074828"

Guselkumab: "Guselkumab" OR "Tremfya" OR "CNTO 1959"

Tildrakizumab: "Tildrakizumab" OR "SCH 900222" OR "MK-3222"

Brazikumab: "Brazikumab" OR "MEDI2070"

(3) Study Design & Methodology Terms

(a) Study Type Filters: "Randomized Controlled Trial"[Publication Type] OR "RCT", "Placebo-controlled" OR "Double-blind" OR "Multicenter Study"

(b) Outcomes & Efficacy Terms: "Clinical remission" OR "Endoscopic remission" OR "Histologic remission", "Response rate" OR "Treatment response", "Safety" OR "Adverse events" OR "Infections" OR "Serious adverse events"

1.2 Search strategy

Refer to the Cochrane Manual to develop a search strategy. The search strategies of different search systems are different. The PubMed search strategy is as follows:

#1 Inflammatory Bowel Diseases"[Mesh]

#2 (Bowel Diseases, Inflammatory[Title/Abstract]) OR (Crohn's Disease[Title/Abstract])) OR (Crohns Disease[Title/Abstract])) OR (Crohn's Enteritis[Title/Abstract])) OR (Inflammatory Bowel Disease 1[Title/Abstract])) OR (Regional Enteritis[Title/Abstract])) OR (Ileocolitis[Title/Abstract])) OR (Ileitis, Terminal[Title/Abstract])) OR (Terminal Ileitis[Title/Abstract])) OR (Ileitis, Regional[Title/Abstract])) OR (Regional Ileitides[Title/Abstract])) OR (Regional Ileitis[Title/Abstract])) OR (Enteritis, Granulomatous[Title/Abstract])) OR (Granulomatous Enteritis[Title/Abstract])) OR (Enteritis, Regional[Title/Abstract])) OR (Colitis, Granulomatous[Title/Abstract])) OR (Granulomatous Colitis[Title/Abstract])) OR (Colitis, Ulcerative[Title/Abstract])) OR (Colitis Gravis[Title/Abstract])) OR (Idiopathic Proctocolitis[Title/Abstract])) OR (Inflammatory Bowel Disease, Ulcerative Colitis Type[Title/Abstract])) OR (Ulcerative Colitis[Title/Abstract])

#3 #1 OR #2

#4 (antagonist[Title/Abstract]) OR (Inhibitor[Title/Abstract])

#5 (Interleukin 23 Subunit p19[Title/Abstract]) OR (IL-23 p19[Title/Abstract])) OR (Interleukin-23 alpha Subunit[Title/Abstract])) OR (alpha Subunit, Interleukin-23[Title/Abstract])) OR (Interleukin 23 alpha Subunit[Title/Abstract])) OR (IL-23p19[Title/Abstract]))) OR ((((((((((((((((((((risankizumab[Title/Abstract]) OR (BI 655066[Title/Abstract])) OR (BI-655066[Title/Abstract])) OR (skyrizi[Title/Abstract])) OR (risankizumab-rzaa[Title/Abstract])) OR (ABBV-066[Title/Abstract])) OR (Brazikumab[Title/Abstract])) OR (MEDI2070[Title/Abstract])) OR (mirikizumab[Title/Abstract])) OR (LY-3074828[Title/Abstract])) OR (LY3074828[Title/Abstract])) OR (guselkumab[Title/Abstract])) OR (Tremfya[Title/Abstract])) OR (CNTO 1959[Title/Abstract])) OR (CNTO-1959[Title/Abstract])) OR (tildrakizumab[Title/Abstract])) OR (SCH 900222[Title/Abstract])) OR (SCH-900222[Title/Abstract])) OR (MK-3222[Title/Abstract])) OR (Ilumya[Title/Abstract])

#6 #4 AND #5

#7 (randomized controlled trial[Publication Type] OR randomized[Title/Abstract] OR placebo[Title/Abstract])

#8 #3 AND #6 AND #7

1.3 Filters and Exclusion Criteria

Language Restriction: English only

Study Type: Only RCTs

Time Frame: No restriction (but prioritizing recent studies)

Population: Adult IBD patients only

Exclusion: Observational studies, case reports, reviews, conference abstracts

1.4 The number of excluded documents and their specific reasons for excluding them.

| Reasons for Exclusion | Number of Documents |
| --- | --- |
| Ineligible study population: Studies including non-UC/CD patients (e.g., other gastrointestinal diseases or healthy volunteers). | 28 |
| Ineligible interventions: Studies not evaluating IL-23 p19 inhibitors (e.g., TNF inhibitors, integrin antagonists, or other biologics). | 6 |
| Inappropriate outcome measures: Studies not reporting primary endpoints such as clinical remission, endoscopic response, or histologic remission. | 2 |
| Non-RCT studies: Observational studies, retrospective analyses, cohort studies, or case reports. | 18 |
| Insufficient follow-up duration or incomplete data: Studies with a follow-up period shorter than 8 weeks or reporting only interim data without final study results. | 1 |

# Supplementary Figures and Tables

## Supplementary Figures


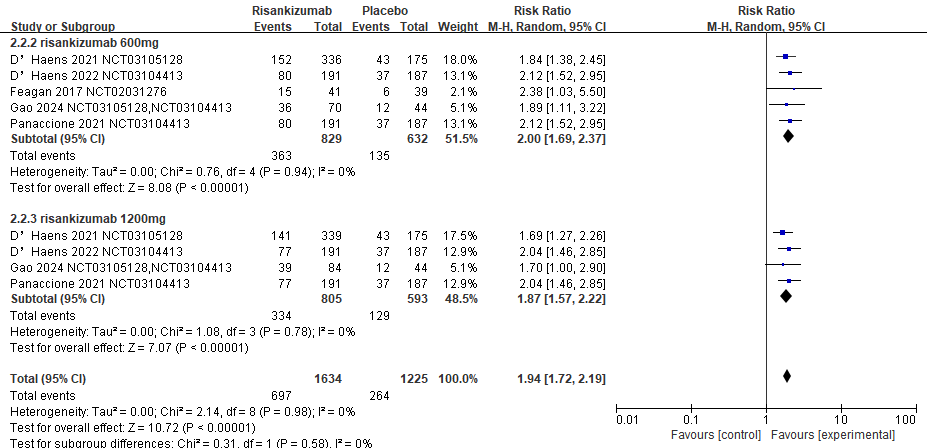


**Supplementary Figure 1.1** Pooled efficacy of different doses of risankizumab for induction of clinical remission in Crohn’s disease.


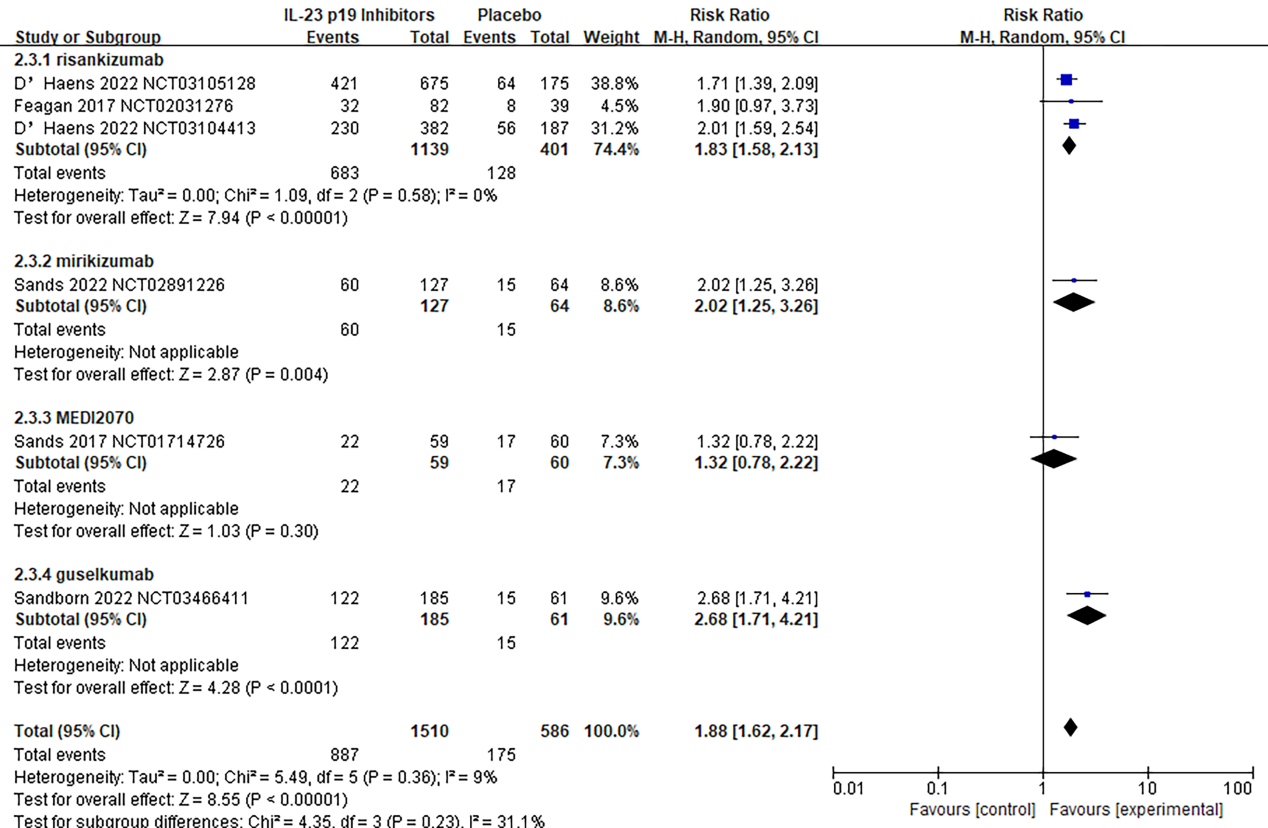


**Supplementary Figure 1.2** Pooled efficacy of IL-23 p19 inhibitors for induction of clinical response in Crohn’s disease.


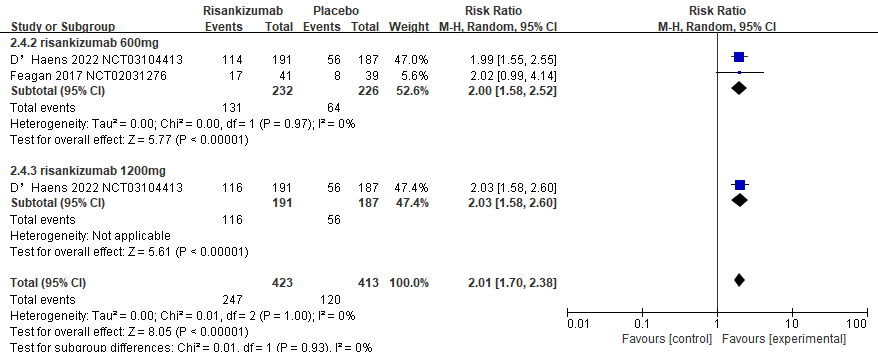


**Supplementary Figure 1.3** Pooled efficacy of different doses of risankizumab for induction of clinical response in Crohn’s disease.


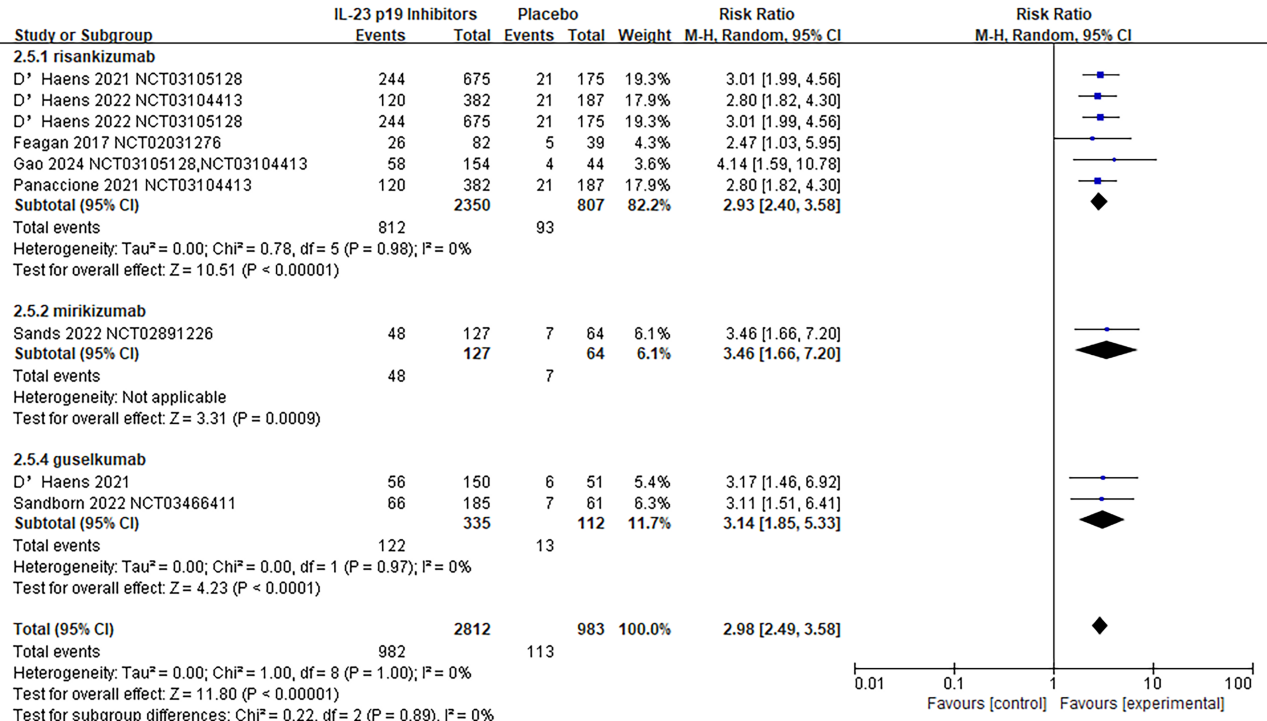


**Supplementary Figure 1.4** Pooled efficacy of IL-23 p19 inhibitors for induction of endoscopic response in Crohn’s disease.


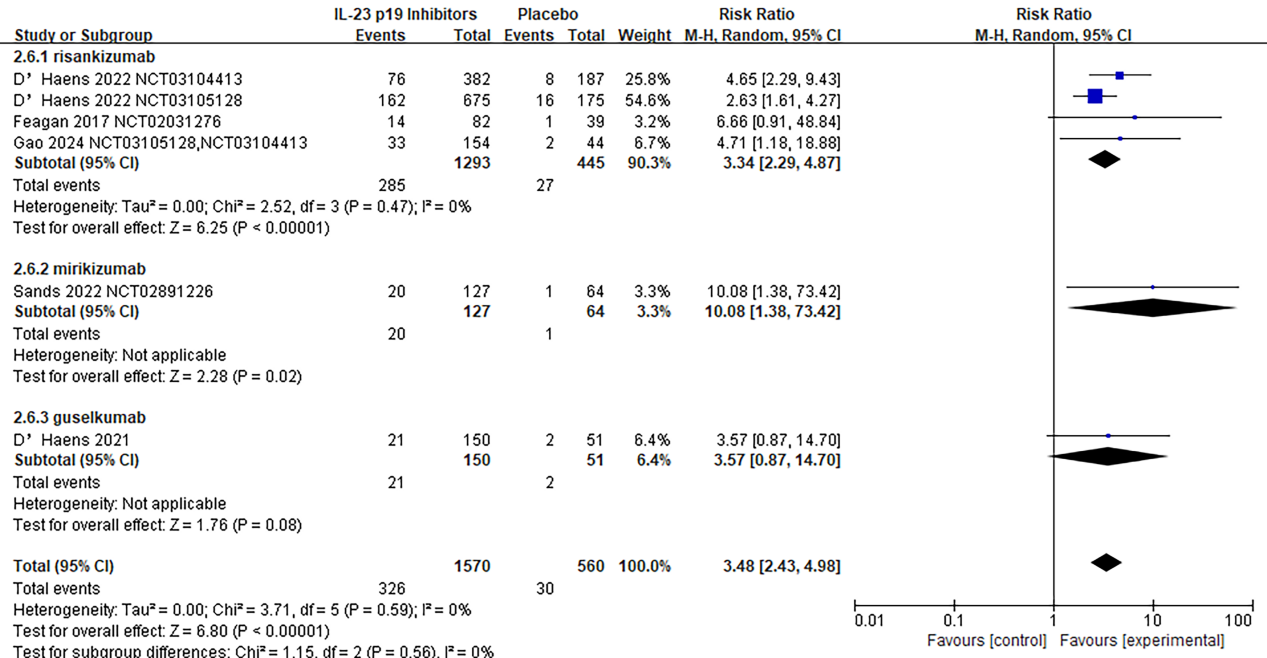


**Supplementary Figure 1.5** Pooled efficacy of IL-23 p19 inhibitors for induction of endoscopic remission in Crohn’s disease.


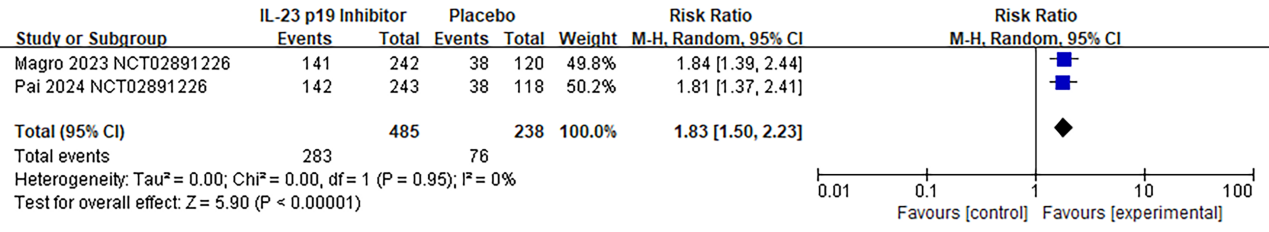


**Supplementary Figure 1.6** Pooled efficacy of IL-23 p19 inhibitors for induction of histological response in Crohn’s disease.


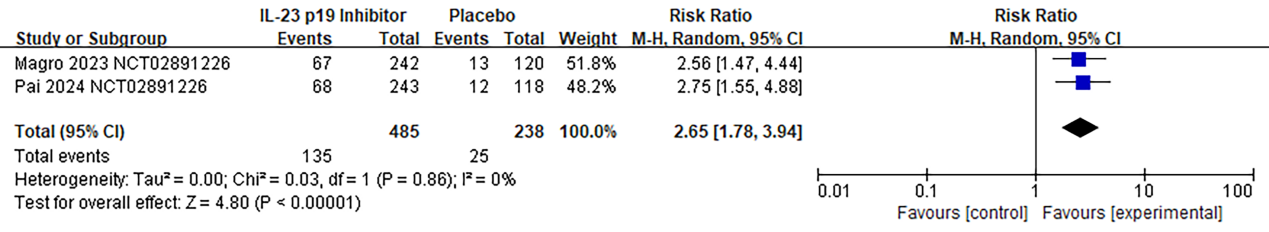


**Supplementary Figure 1.7** Pooled efficacy of IL-23 p19 inhibitors for induction of histological remission in Crohn’s disease.


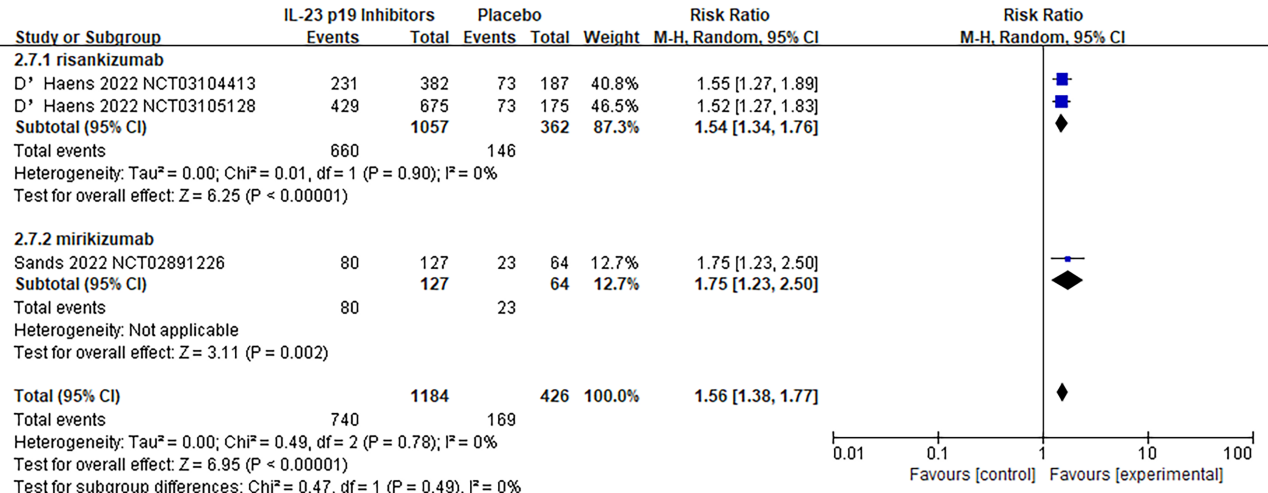


**Supplementary Figure 1.8** Pooled efficacy of IL-23 p19 inhibitors for induction of SF/APS clinical response in Crohn’s disease.


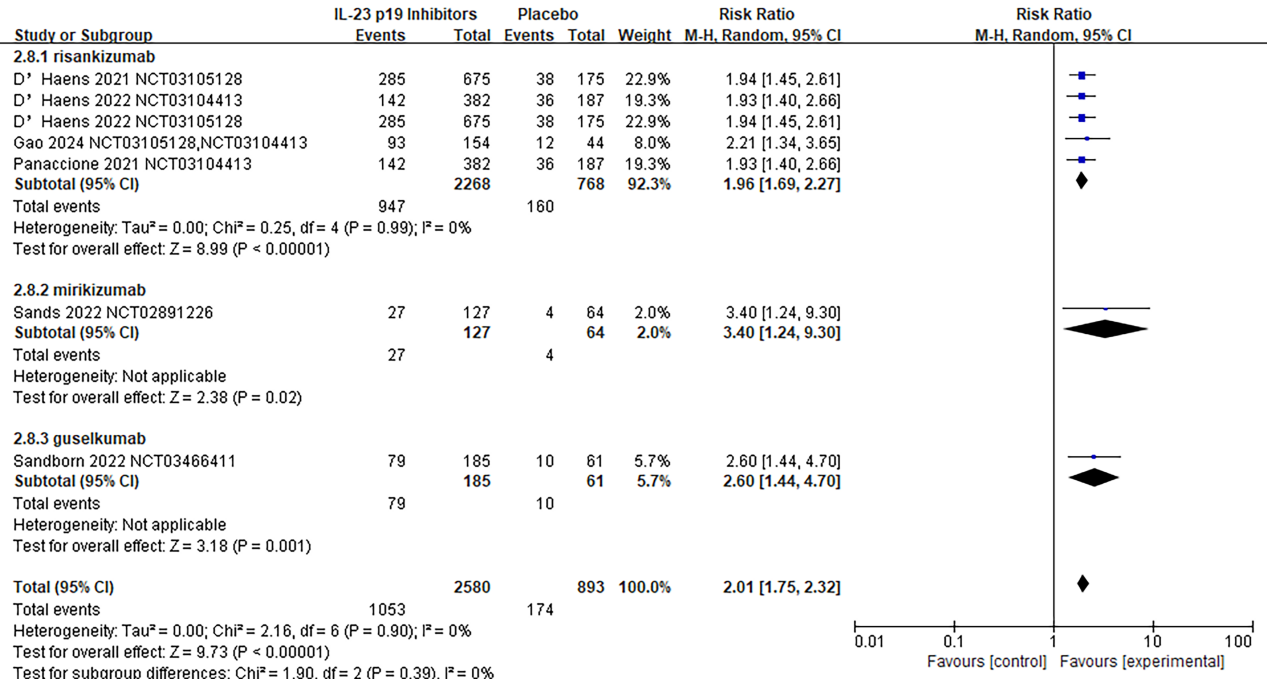


**Supplementary Figure 1.9** Pooled efficacy of IL-23 p19 inhibitors for induction of SF/APS clinical remission in Crohn’s disease.


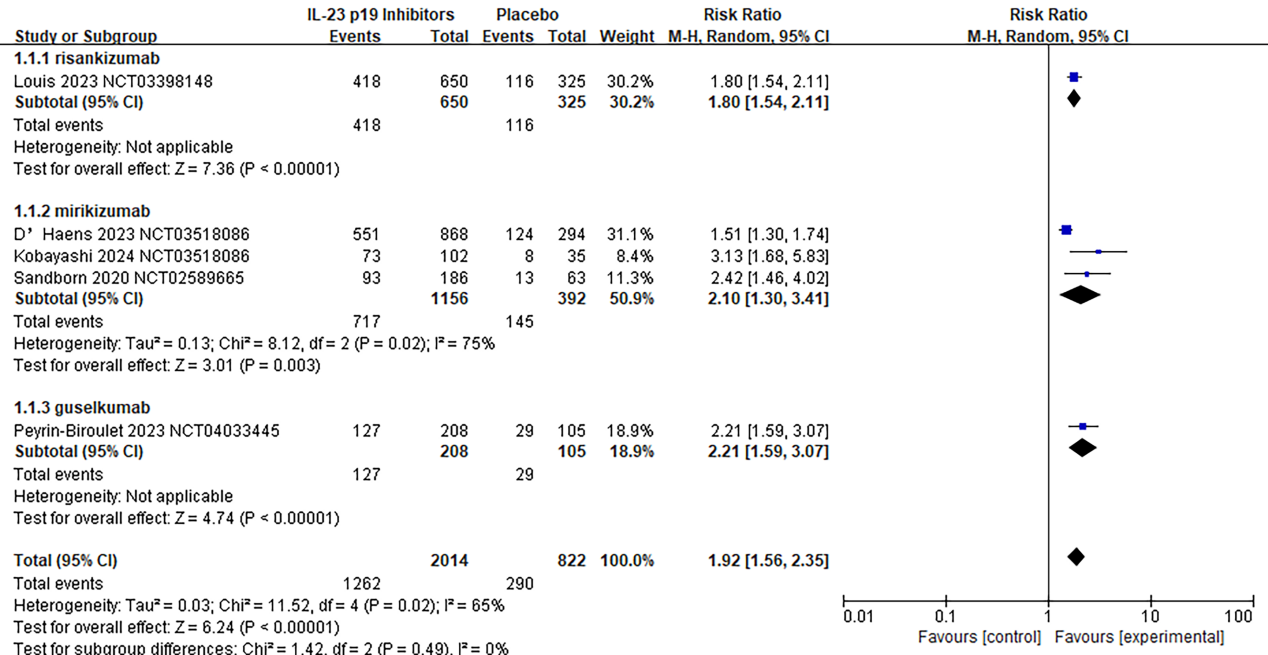


**Supplementary Figure 2.1** Pooled efficacy of IL-23 p19 inhibitors for induction of clinical response in ulcerative colitis.


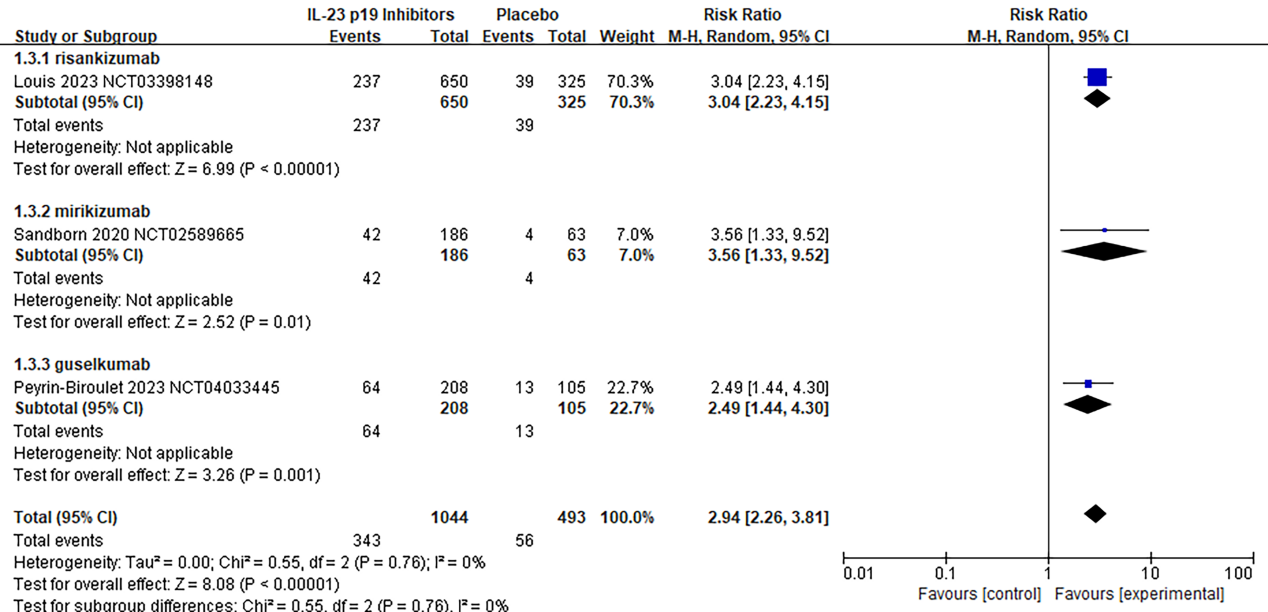


**Supplementary Figure 2.2** Pooled efficacy of IL-23 p19 inhibitors for induction of endoscopic response in ulcerative colitis.


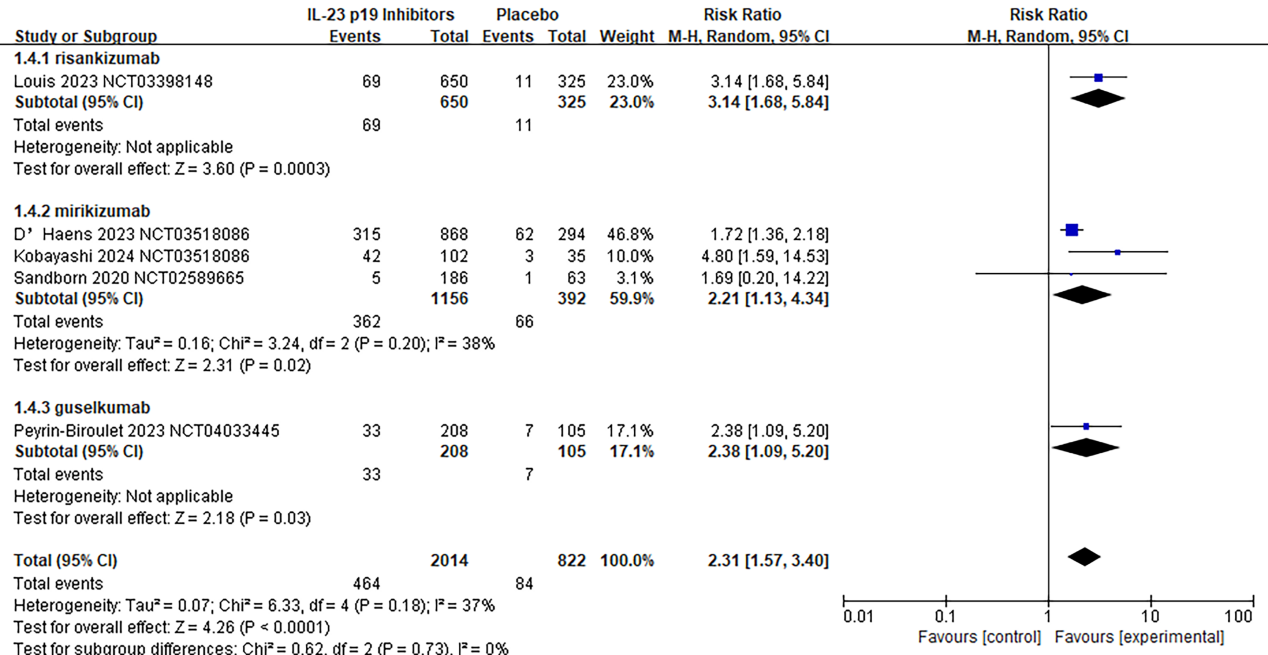


**Supplementary Figure 2.3** Pooled efficacy of IL-23 p19 inhibitors for induction of endoscopic remission in ulcerative colitis.


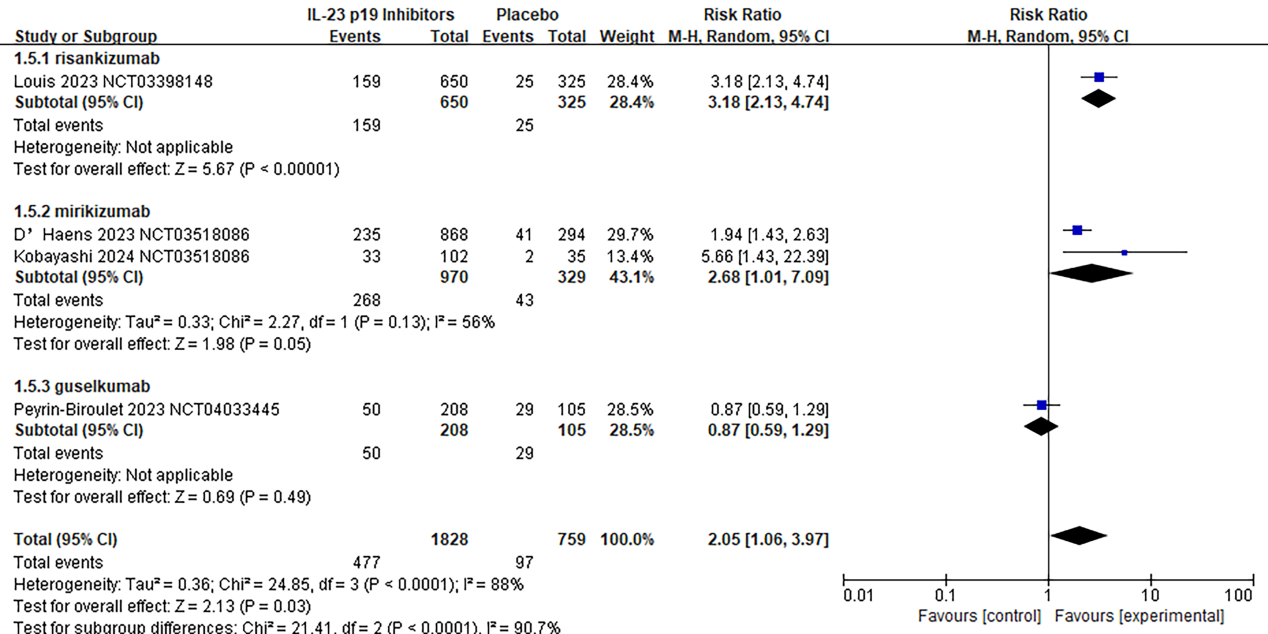


**Supplementary Figure 2.4** Pooled efficacy of IL-23 p19 inhibitors for induction of histologic response in ulcerative colitis.


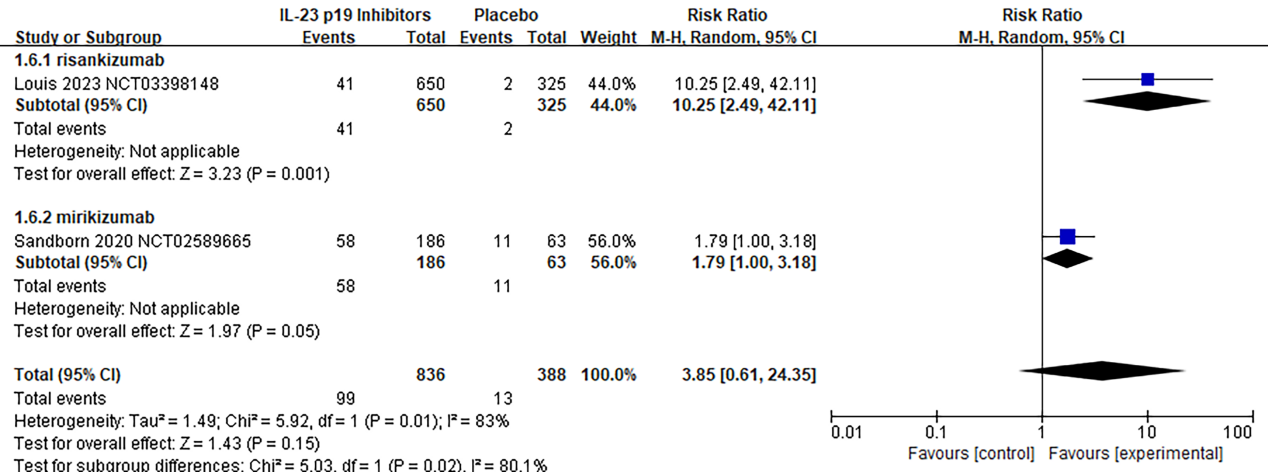


**Supplementary Figure 2.5** Pooled efficacy of IL-23 p19 inhibitors for induction of histologic remission in ulcerative colitis.


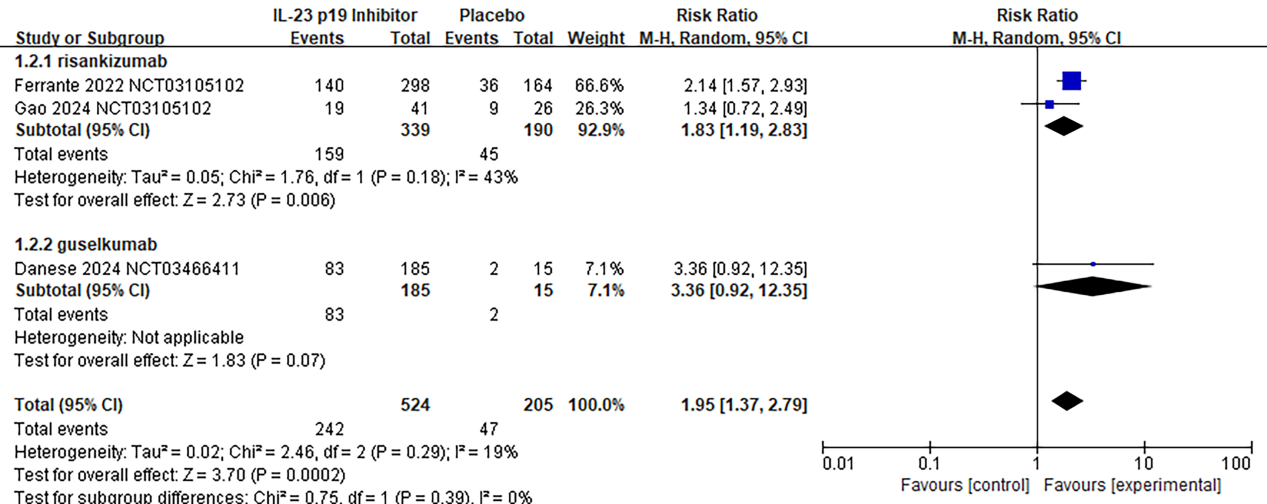


**Supplementary Figure 3** Pooled efficacy of IL-23 p19 inhibitors for maintenance of endoscopic response in Crohn’s disease.


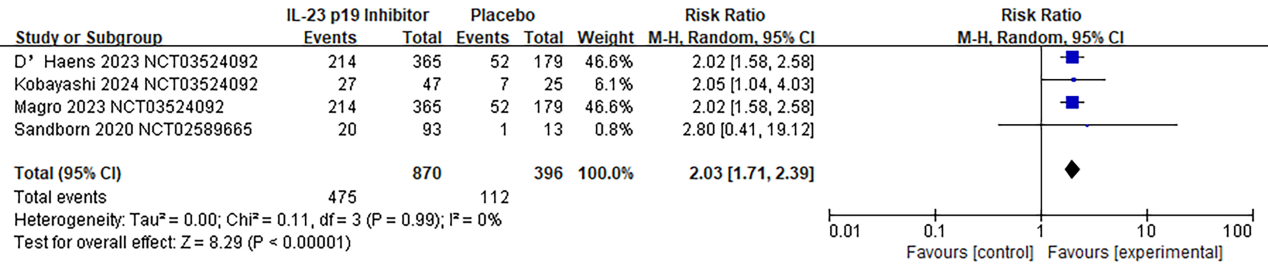


**Supplementary Figure 4** Pooled efficacy of IL-23 p19 inhibitors for maintenance of endoscopic response in ulcerative colitis.


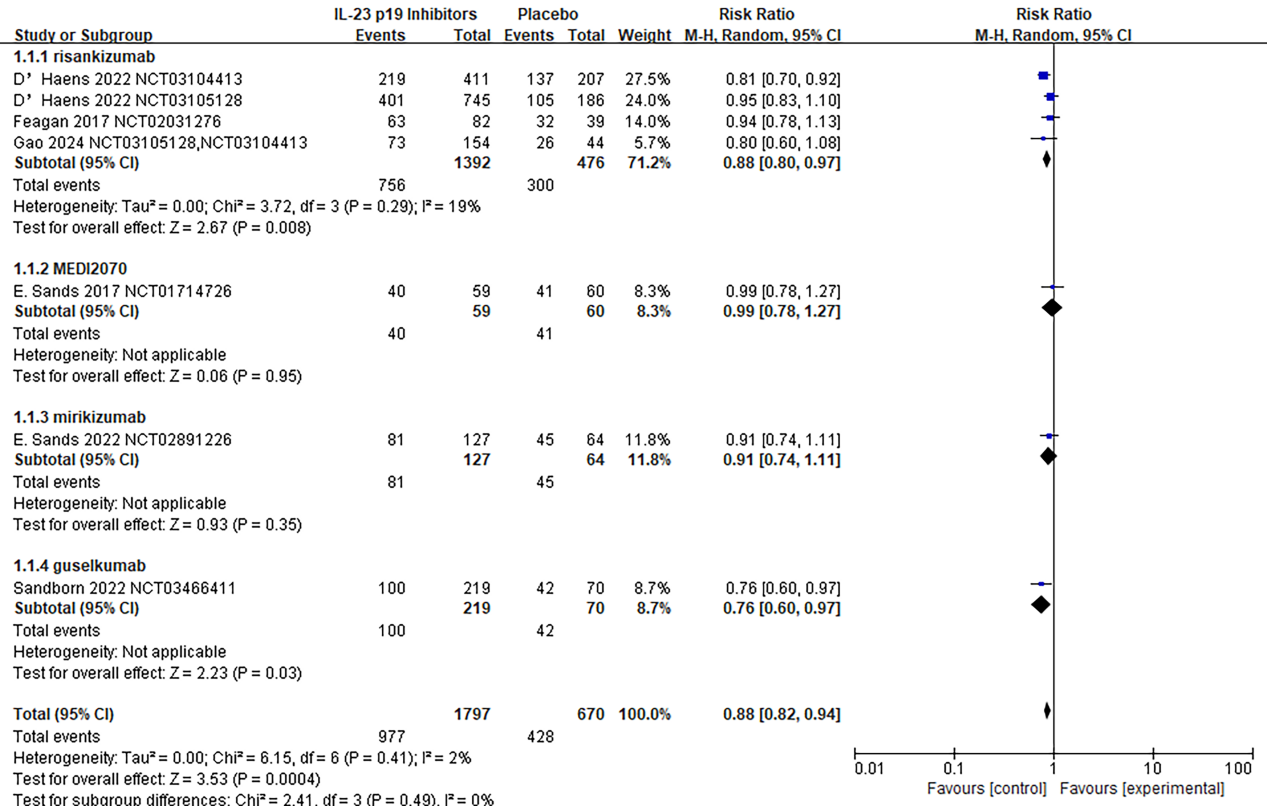


**Supplementary Figure 5.1** Pooled safety of IL-23 p19 inhibitors as Crohn’s disease induction therapy: adverse events.


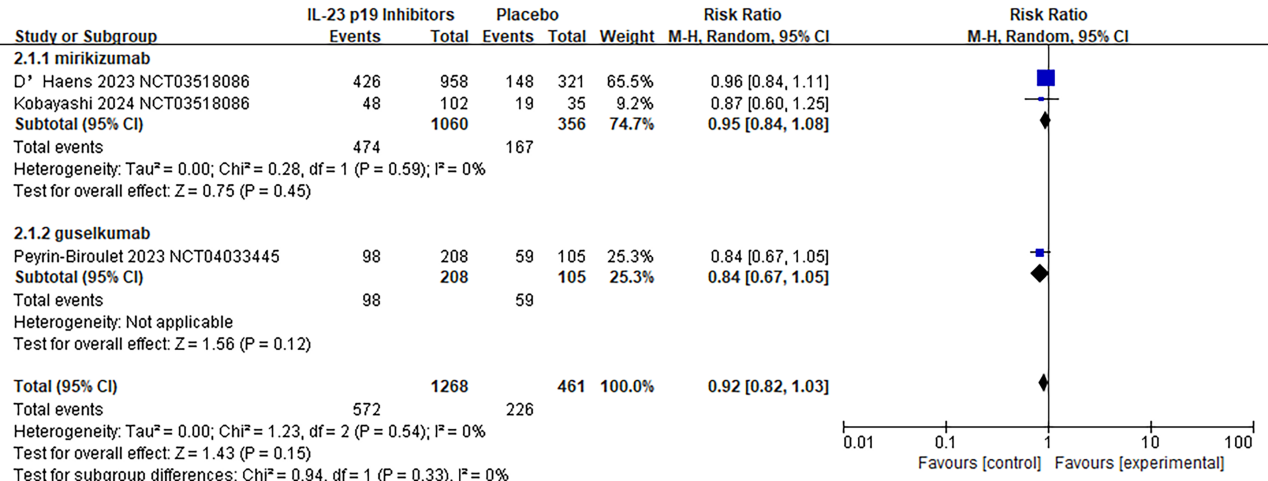


**Supplementary Figure 5.2** Pooled safety of IL-23 p19 inhibitors as ulcerative colitis induction therapy: adverse events.


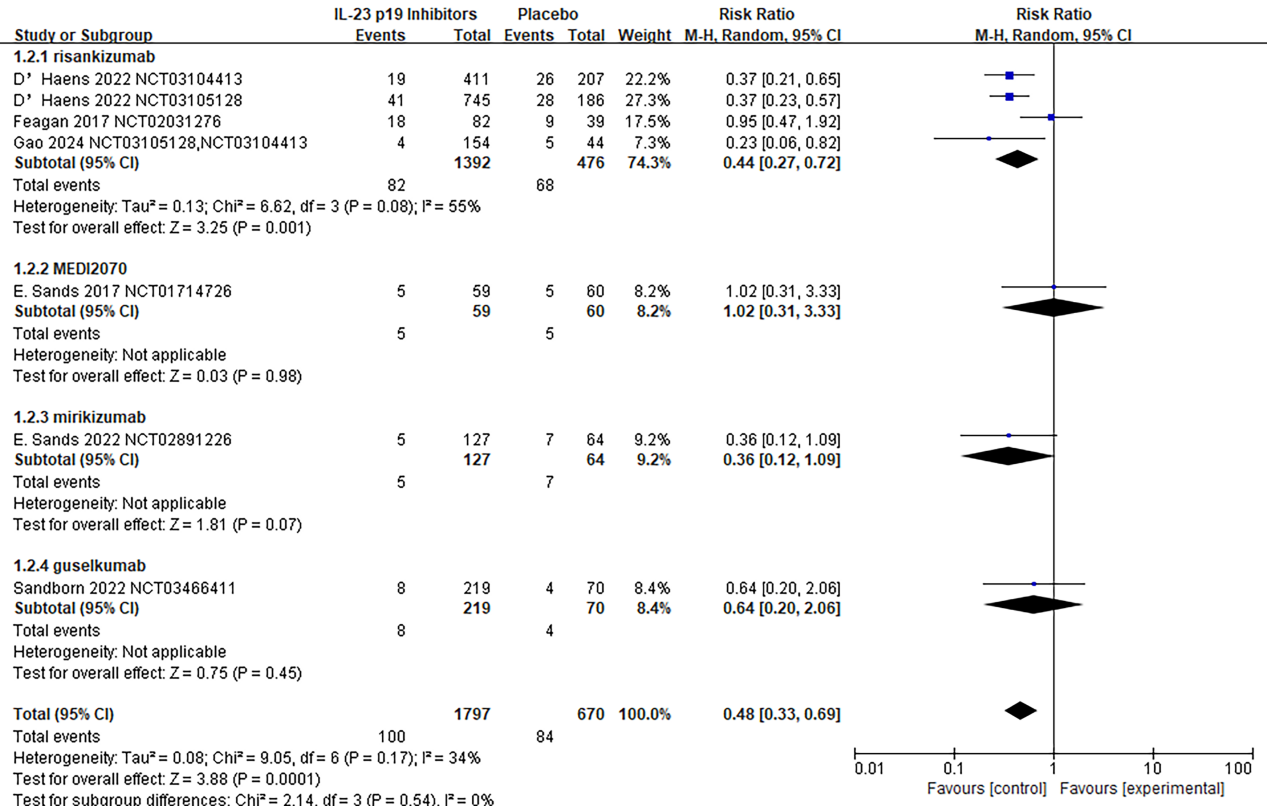


**Supplementary Figure 5.3** Pooled safety of IL-23 p19 inhibitors as Crohn’s disease induction therapy: serious adverse events.


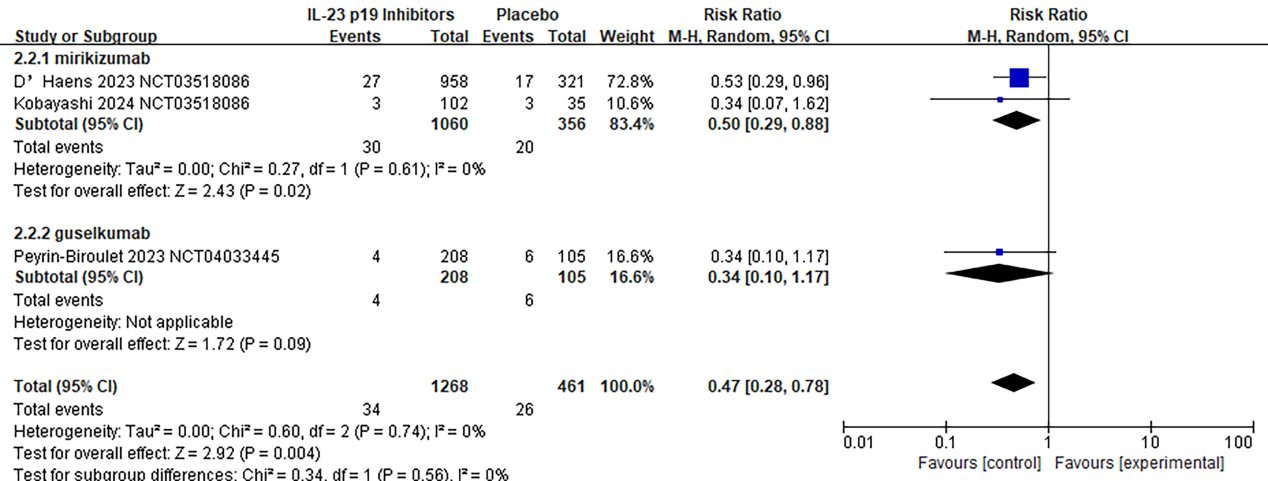


**Supplementary Figure 5.4** Pooled safety of IL-23 p19 inhibitors as ulcerative colitis induction therapy: serious adverse events.


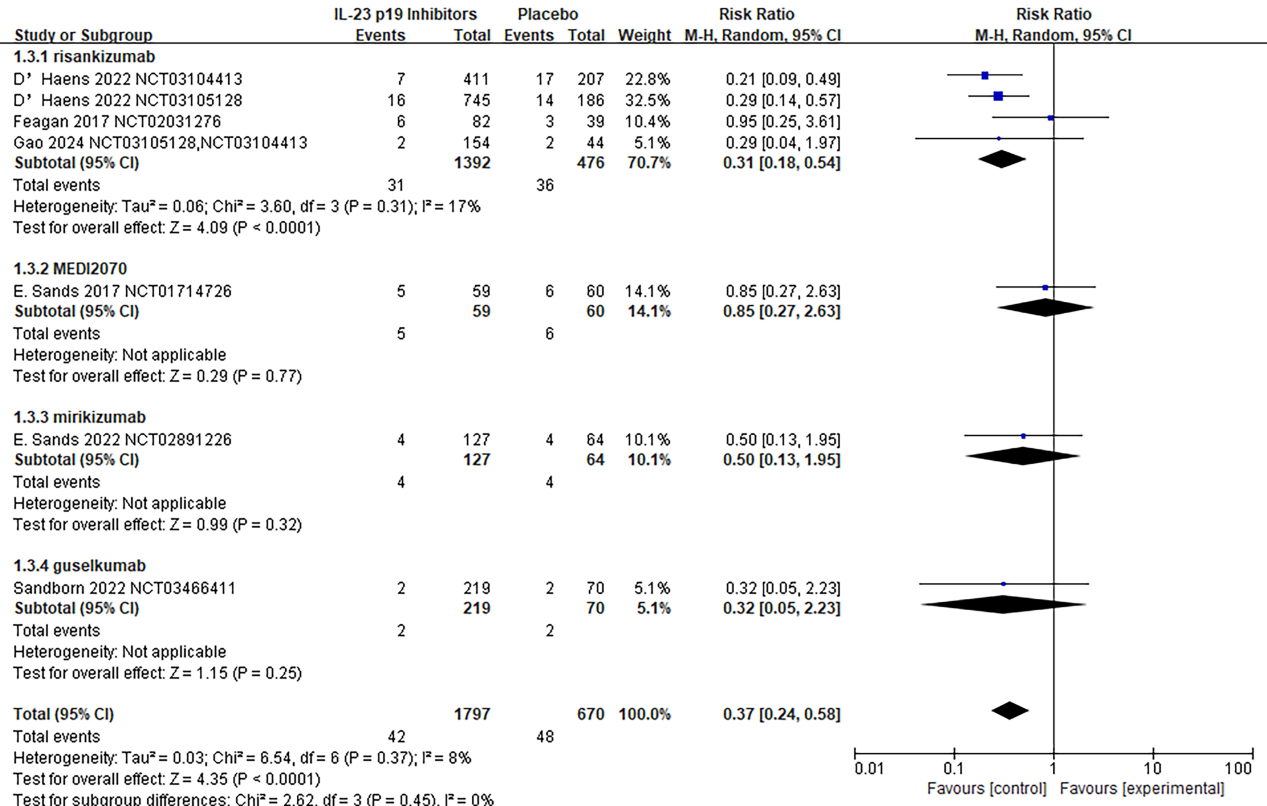


**Supplementary Figure 5.5** Pooled safety of IL-23 p19 inhibitors as Crohn’s disease induction therapy: AE-related discontinuation events.


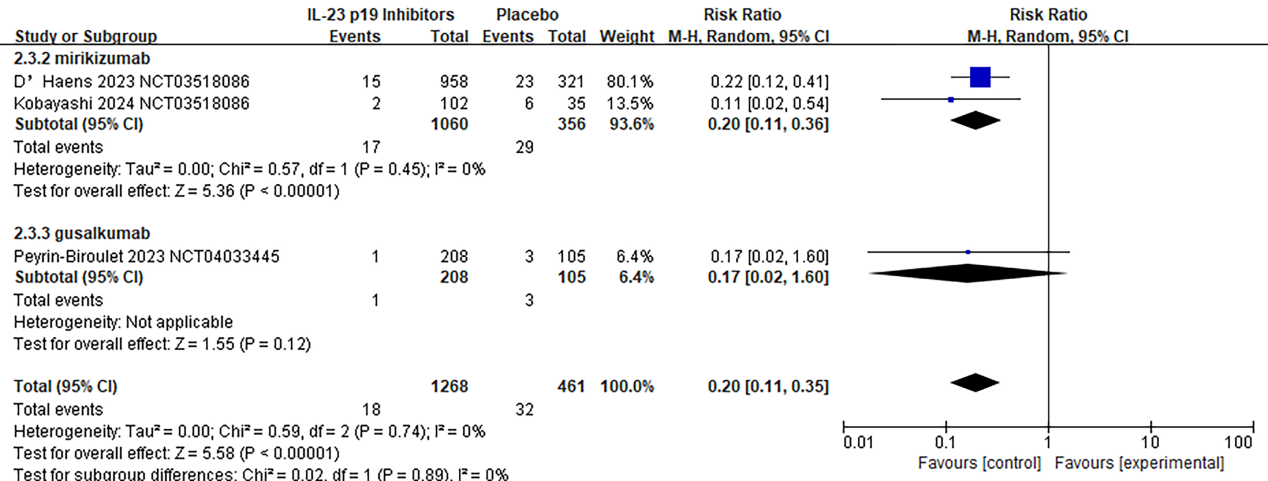


**Supplementary Figure 5.6** Pooled safety of IL-23 p19 inhibitors as ulcerative colitis induction therapy: AE-related discontinuation events.


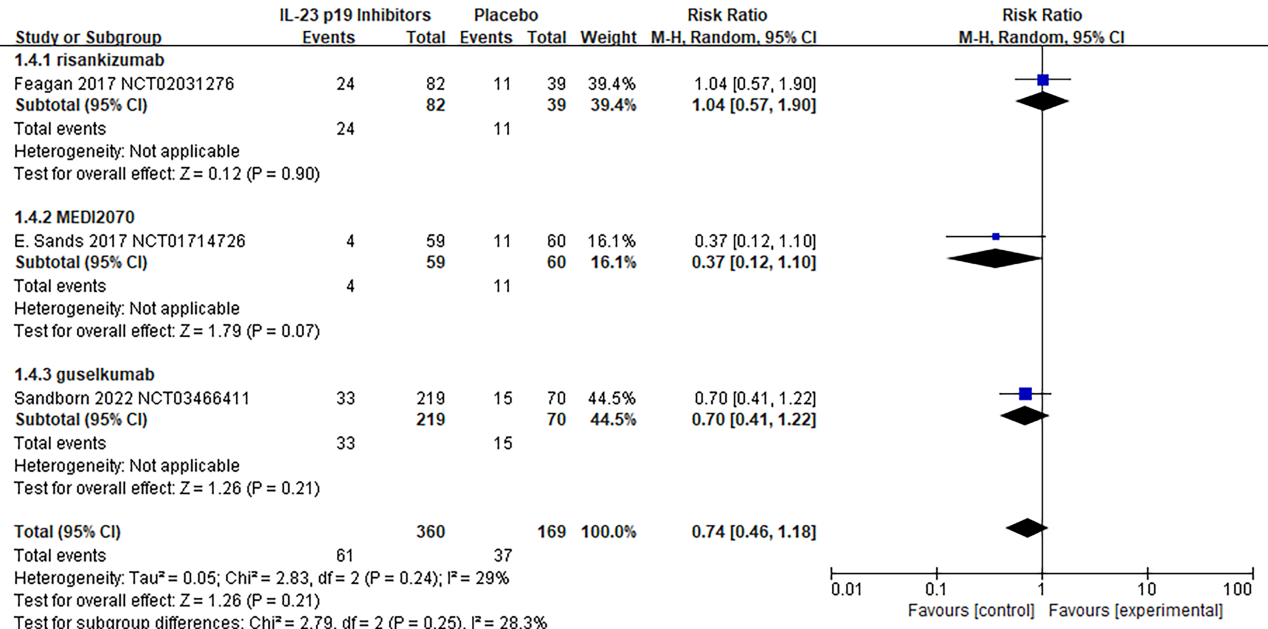


**Supplementary Figure 5.7** Pooled safety of IL-23 p19 inhibitors as Crohn’s disease induction therapy: infection.


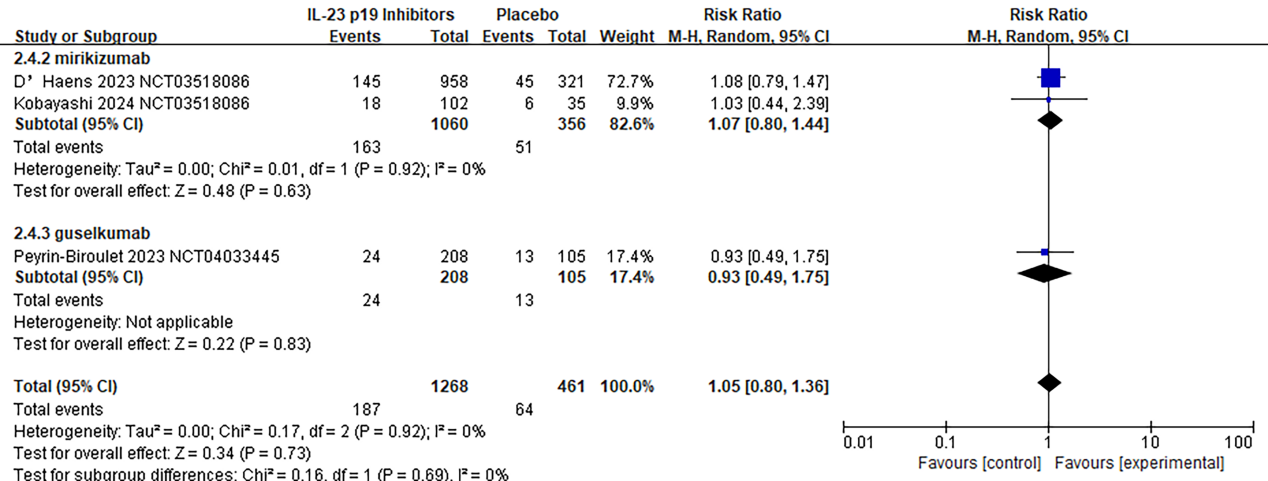


**Supplementary Figure 5.8** Pooled safety of IL-23 p19 inhibitors as ulcerative colitis induction therapy: infection.


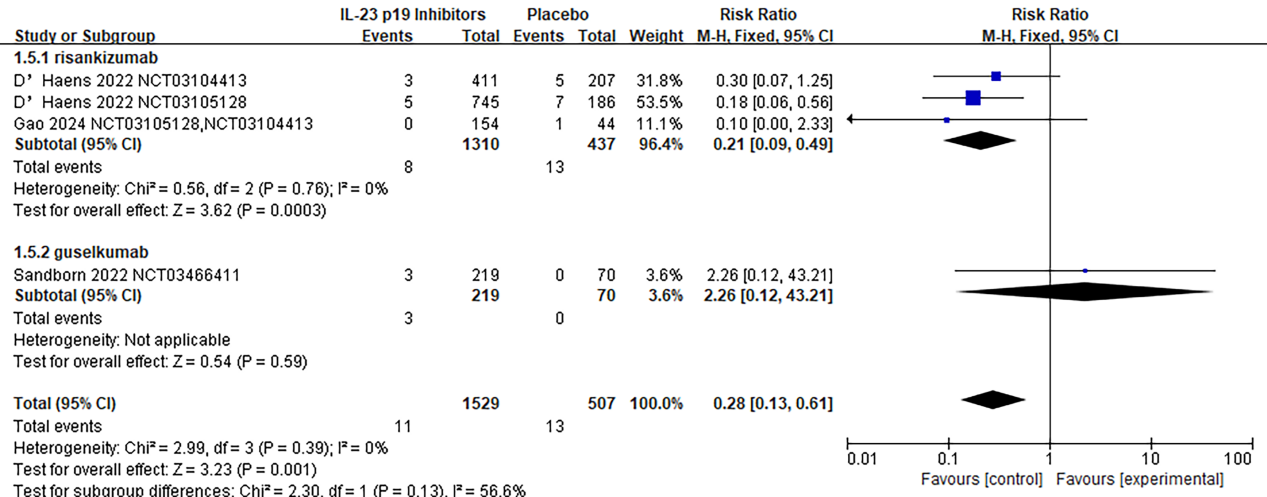


**Supplementary Figure 5.9** Pooled safety of IL-23 p19 inhibitors as Crohn’s disease induction therapy: serious infection.


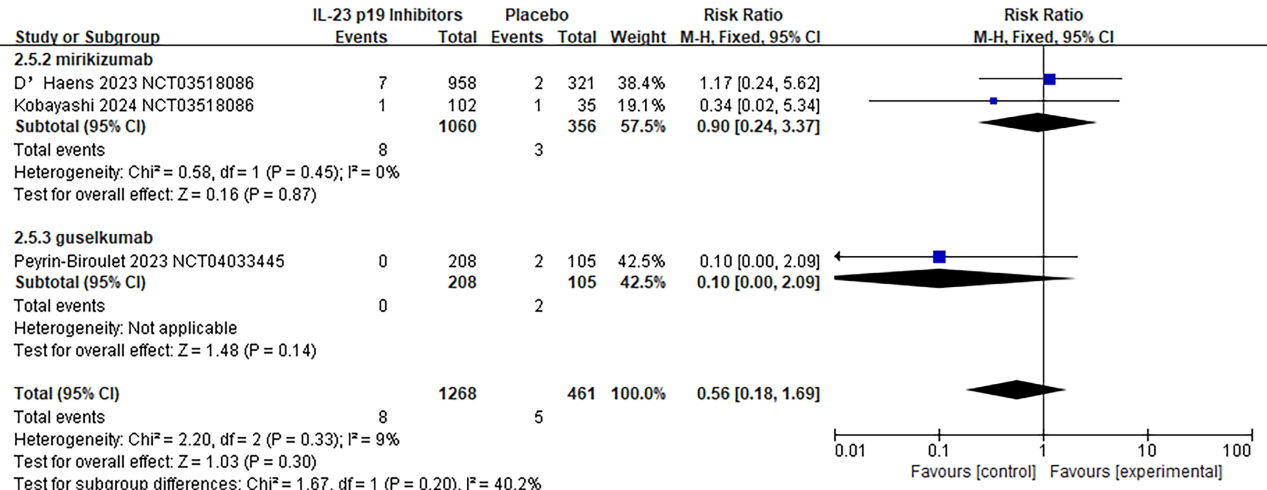


**Supplementary Figure 5.10** Pooled safety of IL-23 p19 inhibitors as ulcerative colitis induction therapy: serious infection.


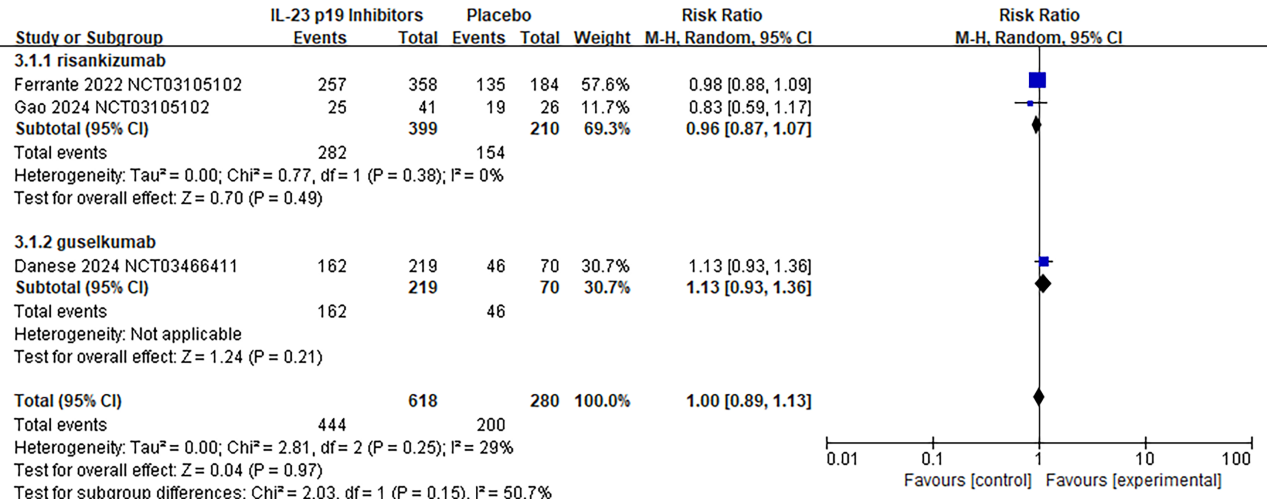


**Supplementary Figure 6.1** Pooled safety of IL-23 p19 inhibitors as Crohn’s disease maintenance therapy: adverse events.


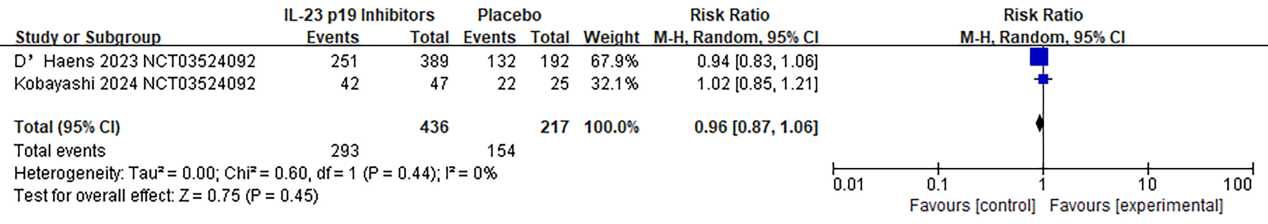


**Supplementary Figure 6.2** Pooled safety of IL-23 p19 inhibitors as ulcerative colitis maintenance therapy: adverse events.


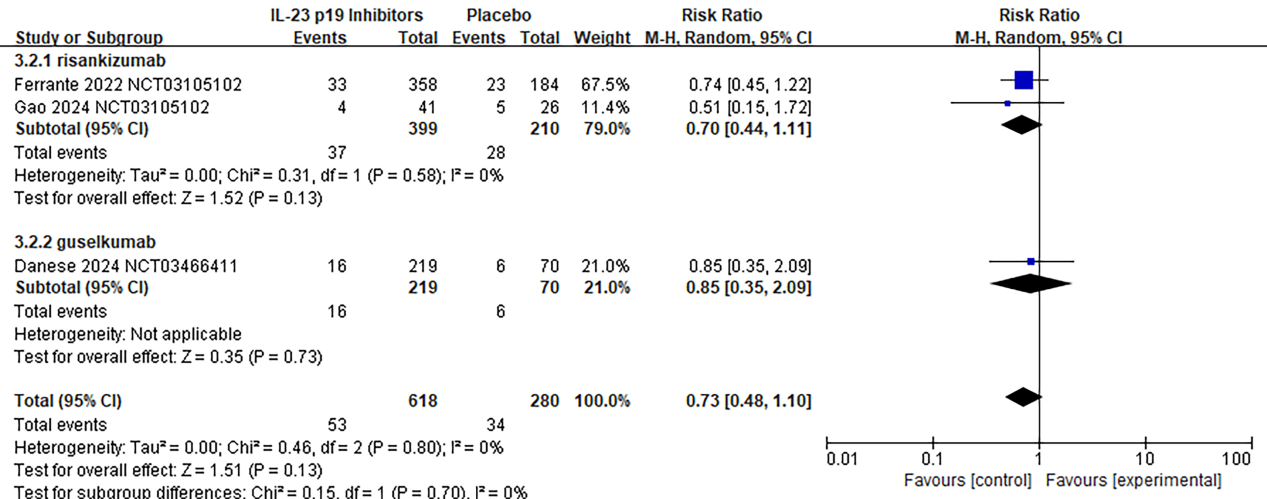


**Supplementary Figure 6.3** Pooled safety of IL-23 p19 inhibitors as Crohn’s disease maintenance therapy: serious adverse events.


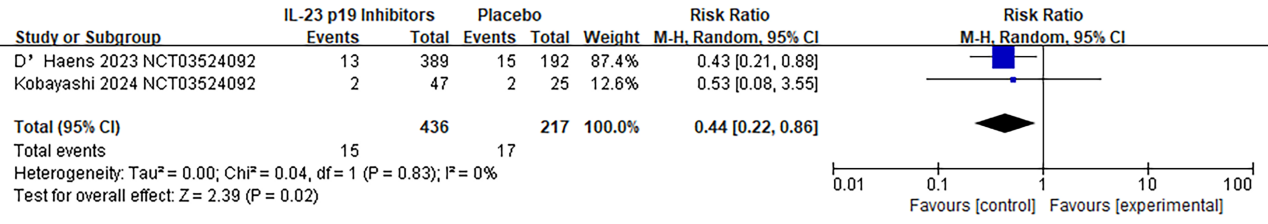


**Supplementary Figure 6.4** Pooled safety of IL-23 p19 inhibitors as ulcerative colitis maintenance therapy: serious adverse events.


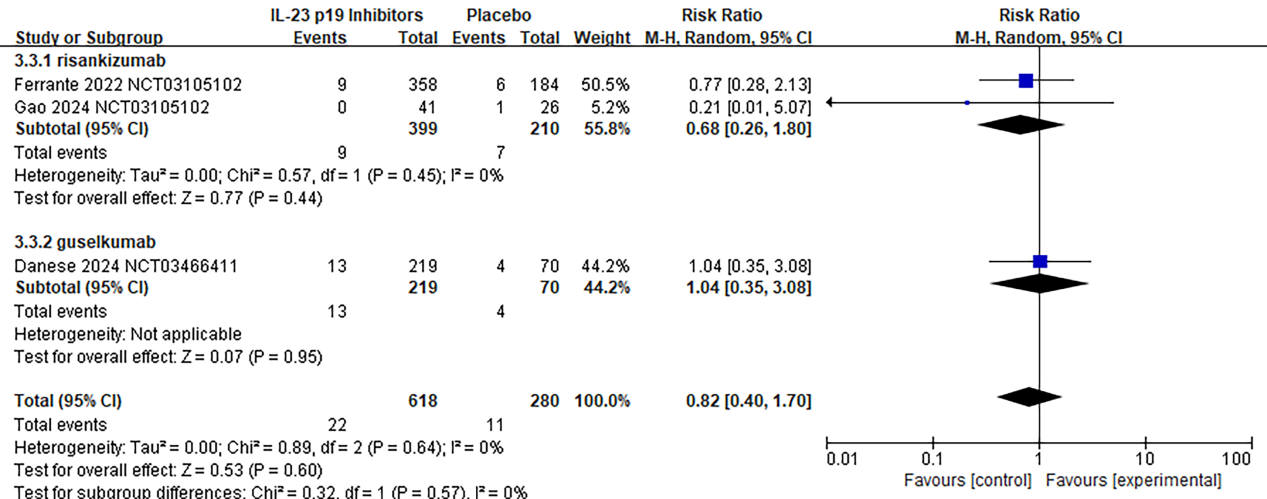


**Supplementary Figure 6.5** Pooled safety of IL-23 p19 inhibitors as Crohn’s disease maintenance therapy: AE-related discontinuation events.


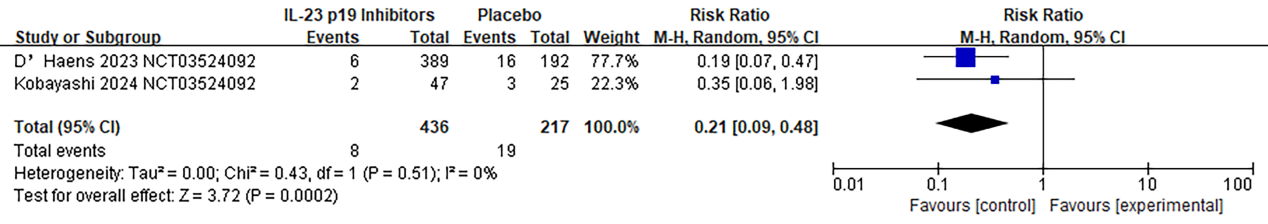


**Supplementary Figure 6.6** Pooled safety of IL-23 p19 inhibitors as ulcerative colitis maintenance therapy: AE-related discontinuation events.


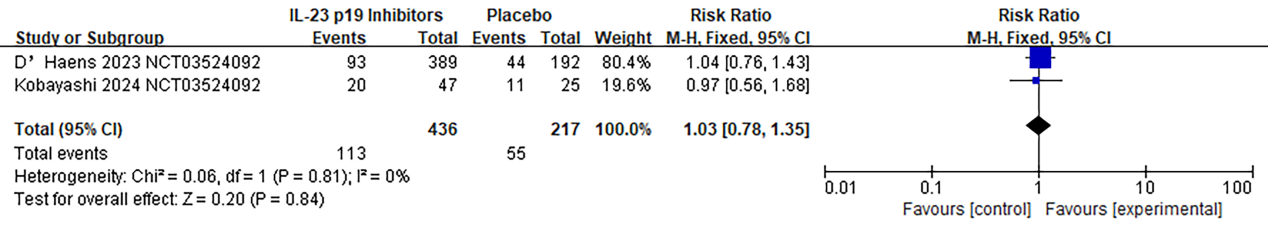


**Supplementary Figure 6.7** Pooled safety of IL-23 p19 inhibitors as ulcerative colitis maintenance therapy: infection.


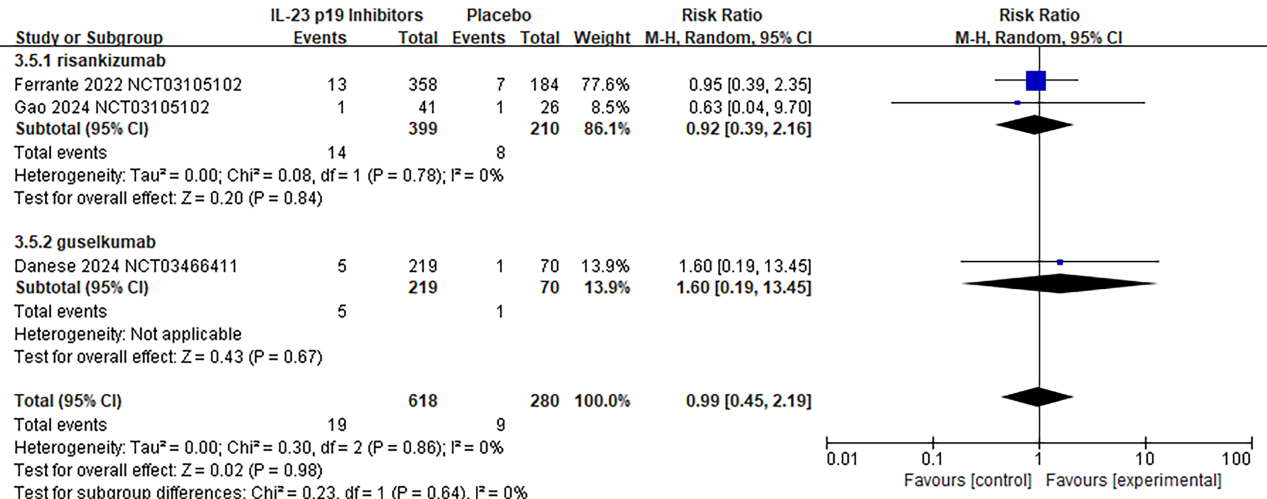


**Supplementary Figure 6.8** Pooled safety of IL-23 p19 inhibitors as Crohn’s disease maintenance therapy: serious infection.


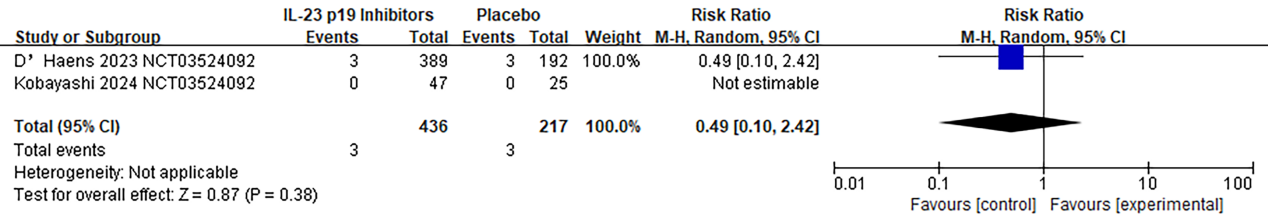


**Supplementary Figure 6.9** Pooled safety of IL-23 p19 inhibitors as ulcerative colitis maintenance therapy: serious infection.

## Supplementary Tables

| **NO** | **YEAR** | **Title** | **Author** | **Country** |
| --- | --- | --- | --- | --- |
| 1 | 2022 | Risankizumab as induction therapy for Crohn’s disease: results from the phase 3 ADVANCE and MOTIVATE induction trials | Geert D’Haens, Remo Panaccione | Multi-country |
| 2 | 2022 | Risankizumab as maintenance therapy for moderately to severely active Crohn’s disease: results from the multicentre, randomised, double-blind, placebo-controlled, withdrawal phase 3 FORTIFY maintenance trial | Marc Ferrante, Remo Panaccione | Multi-country |
| 3 | 2017 | Induction therapy with the selective interleukin-23 inhibitor risankizumab in patients with moderate-to-severe Crohn’s disease: a randomised, double-blind, placebo-controlled phase 2 study | Brian G Feagan | Multi-country |
| 4 | 2017 | Efficacy and Safety of MEDI2070, an Antibody Against Interleukin 23, in Patients With Moderate to Severe Crohn’s Disease: A Phase 2a Study | Bruce E. Sands | Multi-country |
| 5 | 2022 | Efficacy and Safety of Mirikizumab in a Randomized Phase 2 Study of Patients With Crohn's Disease | Bruce E. Sands | Multi-country |
| 6 | 2022 | Guselkumab for the Treatment of Crohn’s Disease: Induction Results From the Phase 2 GALAXI-1 Study | William J. Sandborn | Multi-country |
| 7 | 2020 | Efficacy and Safety of Mirikizumab in a Randomized Phase 2 Study of Patients With Ulcerative Colitis | William J. Sandborn | Multi-country |
| 8 | 2023 | Mirikizumab as Induction and Maintenance Therapy for Ulcerative Colitis | Geert D’Haens | Multi-country |
| 9 | 2023 | Guselkumab in Patients With Moderately to Severely Active Ulcerative Colitis: QUASAR Phase 2b Induction Study | Laurent Peyrin-Biroulet | Multi-country |
| 10 | 2023 | Resolving Histological Inflammation in Ulcerative Colitis With Mirikizumab in the LUCENT Induction and Maintenance Trial Programmes | Fernando Magro | Multi-country |
| 11 | 2024 | Efficacy and safety of 48 weeks of guselkumab for patients with Crohn’s disease: maintenance results from the phase 2, randomised, double-blind GALAXI-1 trial | Silvio Danese | Multi-country |
| 12 | 2023 | Effects of Mirikizumab on Histologic Resolution of Crohn’s Disease in a Randomized Controlled Phase 2 Trial | Fernando Magro | Multi-country |
| 13 | 2024 | Efficacy and safety of mirikizumab as induction and maintenance therapy for Japanese patients with moderately to severely active ulcerative colitis: a subgroup analysis of the global phase 3 LUCENT-1 and LUCENT-2 studies | Taku Kobayashi | Japan |
| 14 | 2023 | Risankizumab Induction Therapy in Patients With Moderately to Severely Active Ulcerative Colitis: Efficacy and Safety in the Randomized Phase 3 INSPIRE Study | Edouard Louis | - |
| 15 | 2024 | Efficacy and safety of risankizumab for Crohn's disease in patients from Asian countries: a post hoc subanalysis of the global phase 3 ADVANCE, MOTIVATE, and FORTIFY studies | Xiang Gao | Multi-country |
| 16 | 2021 | 775a Risankizumab Induction Therapy in Patients With Moderate-to-Severe Crohn’s Disease with Intolerance or Inadequate Response to Conventional and/or Biologic Therapy: Results from the Phase 3 ADVANCE Study | Geert R. D’Haens | Multi-country |
| 17 | 2021 | S754 Risankizumab as Induction Therapy in Patients with Moderately to Severely Active Crohn’s Disease Who Failed 1 vs >1 Prior Biologic Treatment: Results From the MOTIVATE Study | Remo Panaccione | Multi-country |
| 18 | 2021 | 455 The effect of guselkumab induction therapy on endoscopic outcome measures in patients with moderately to severely active crohn's disease: week 12 results from the phase 2 galaxi 1 study | Geert D’Haens | Multi-country |
| 19 | 2024 | P395 Impact of mirikizumab therapy on histologic measures of intestinal inflammation in a Phase 2 study of patients with moderately to severely active Crohn’s disease | R. Pai | Multi-country |

**Supplementary Table 1.** Included studies.

| Endpoint | Definition |
| --- | --- |
| Clinical response | A modified Mayo stool-frequency subscore of 0 (on a scale from 0 to 3, with higher scores indicating higher frequency) or a stool-frequency subscore of 1 with a decrease of at least 1 point from baseline, a rectal-bleeding subscore of 0, and an endoscopic subscore of 0 or 1 (excluding friability). |
| Clinical remission | Decreases of ≥2 points and ≥30% from baseline in the modified Mayo score, plus a rectal-bleeding subscore of 0 or 1 or a decrease of ≥1 point from baseline |
| Endoscopic response | An endoscopic subscore of 0 or 1 [excluding friability] |
| Endoscopic remission | Achieving a Mayo endoscopic subscore of 0 |
| Histologic response | Geboes score ≤3.1: neutrophil infiltration in <5% of crypts; no crypt destruction; and no erosions, ulcerations, or granulation tissue |
| Histologic remission | Geboes score ≤2.0: no lamina propria neutrophils; no neutrophils in the surface or crypt epithelium; no crypt destruction; and no erosions, ulcerations, or granulation tissue |

**Supplementary Table 2.** Ulcerative colitis outcome measures included in the studies.

| Endpoint | Definition |
| --- | --- |
| Clinical response | Reduction of CDAI ≥100 points from baseline. |
| Clinical remission | CDAI less than 150. |
| SF/APS clinical response | Stool frequency and abdominal pain score clinical response, mean daily bowel movement frequency and abdominal pain scores were reduced by ≥30%, both non-inferior to baseline |
| SF/APS clinical remission | Stool frequency and abdominal pain score clinical remission, mean daily liquid or very soft stool frequency of 2.8 or less and not worse than baseline, plus mean daily abdominal pain score of 1 or less and not worse than baseline. |
| Endoscopic response | A decrease in SES-CD of more than 50% from baseline, or for patients with isolated ileal disease and a baseline SES-CD of 4, at least a 2-point reduction from baseline. |
| Endoscopic remission | SES-CD ≤4, ≥2-point reduction vs baseline, and no subscore ≥1 in any variable. |
| Histologic response | No epithelial neutrophils or epithelial damage, or ≥50% decrease of a-GHAS, m-GHAS, or RHI from baseline in the histologic analysis population. |
| Histologic remission | The absence of mucosal neutrophils or epithelial damage. |

**Supplementary** **Table 3.** Crohn's disease outcome measures included in the studies.

| Study reference | Therapy period & time to primary outcome | Treatment Arms (n) | CDAI Clinical Response, n (%) | SF/APS Clinical Response, n (%) | CDAI Clinical Remission, n (%) | SF/APS Clinical Remission, n (%) | Endoscopic Response, n (%) | Endoscopic Remission, n (%) | Histologic Response, n (%) | Histologic Remission, n (%) |
| --- | --- | --- | --- | --- | --- | --- | --- | --- | --- | --- |
| 1. D’Haens 2022   (NCT03105128) | Induction  12 weeks | Risankizumab 600 mg IV (336)  Risankizumab 1200 mg IV (339)  Placebo (175) | 201(60%)  220(65%)  64(37%) | 211(63%)  218(64%)  73(42%) | 152(45.2%)  141(41.6%)  43(24.6%) | 146(43.5%)  139(41.0%)  38(21.7%) | 135(40.3%)  109(32.1%)  21(12.0%) | 81(24%)  81(24%)  16(9%) | NR | NR |
| 1. D’Haens 2022   (NCT03104413) | Induction  12 weeks | Risankizumab 600 mg IV (191)  Risankizumab 1200 mg IV (191)  Placebo (187) | 114(60%)  116(61%)  56(30%) | 118(62%)  113(59%)  73(39%) | 80(42.0%)  77(40.3%)  37(19.8%) | 66(34.6%)  76(39.8%)  36(19.3%) | 55(28.8%)  65(34.2%)  21(11.2%) | 37(19%)  39(20%)  8(4%) | NR | NR |
| 1. Ferrante 2022   (NCT03105102) | Maintenance  52 weeks | Risankizumab 600 mg SC (157)  Risankizumab 1200 mg SC (141)  Placebo (164) | 105(67%)  87(62%)  79(48%) | 97(62%)  84(59%)  81(49%) | 87(55.4%)  74(52.5%)  67(40.9%) | 73(46.5%)  73(51.8%)  65(39.6) | 74(47.1%)  66(46.8%)  36(22.0%) | 47(30%)  55(39%)  21(13%) | NR | 38(24%)  43(31%)  17(10%) |
| 1. Feagan 2017   (NCT02031276) | Induction  12 weeks | Risankizumab 200 mg IV (41)  Risankizumab 600 mg IV (41)  Placebo (39) | 15(36.6%)  17(41.5%)  8(20.5%) | NR | 10(24.4%)  15(36.6%)  6(15.4%) | NR | 11(27%)  15(37%)  5(13%) | 6(15%)  8(20%)  1(3%) | NR | NR |
| 1. Gao 2024   (NCT03105128)  (NCT03104413) | Induction  12 weeks | Risankizumab 600 mg IV (70)  Risankizumab 1200 mg IV (84)  Placebo (44) | NR | NR | 36(51.4%)  39(46.5%)  12(27.3%) | 43(61.4%)  50(59.5%)  12(27.3%) | 28(40.0%)  30(35.8%)  4(9.1%) | 16(22.9%)  17(20.2%)  2(4.5%) | NR | NR |
| 1. Gao 2024   (NCT03105102) | Maintenance  52 weeks | Risankizumab 180 mg IV (21)  Risankizumab 360 mg IV (20)  Placebo (26) | NR | NR | 11(52.4%)  13(65.0%)  14(53.8%) | 12(57.1%)  15(75.0%)  14(53.8%) | 11(52.4%)  8(40.0%)  9(34.6%) | 7(33.3%)  7(35.0%)  5(19.2%) | NR | NR |
| 1. D’Haens 2021   (NCT03105128) | Induction  12 weeks | Risankizumab 600 mg IV (336)  Risankizumab 1200 mg IV (339)  Placebo (175) | NR | NR | 152 (45.2%)  141 (41.6%)  43 (24.6%) | 146 (43.5%)  139 (41.0%)  38 (21.7%) | 135 (40.3%)  109 (32.1%)  21 (12.0%) | NR | NR | NR |
| 1. Panaccione 2021   (NCT03104413) | Induction  12 weeks | Risankizumab 600 mg IV (191)  Risankizumab 1200 mg IV (191)  Placebo (187) | NR | NR | 80(42.0%)  77(40.3%)  37(19.8%) | 66(34.6%)  76(39.8%)  36(19.3%) | 55(28.8%)  65(34.2%)  21(11.2%) | NR | NR | NR |
| 1. E. Sands 2022   (NCT02891226) | Induction  12 weeks | Mirikizumab 200 mg IV (31)  Mirikizumab 600 mg IV (32)  Mirikizumab 1000 mg IV (64)  Placebo (64) | 15(48.4%)  18(56.3%)  27(42.2%)  15(48.4%) | 19(61.3%)  22(68.8%)  39(60.9%)  23(35.9%) | 5(16.1%)  13(40.6%)  17(26.6%)  6(9.4%) | 4(12.9%)  9(28.1%)  14(21.9%)  4(6.3%) | 8(25.8%)  12(37.5%)  28(43.8%)  7(10.9%) | 2(6.5%)  5(15.6%)  13(20.3%)  1(1.6%) | NR | NR |
| 1. Magro 2023   (NCT02891226) | Induction  12 weeks | Mirikizumab 200 mg IV (65)  Mirikizumab 600 mg IV (63)  Mirikizumab 1000 mg IV (114)  Placebo (120) | NR | NR | NR | NR | NR | NR | 34(52.3%)  30(47.6%)  77(67.5%)  38(31.7%) | 15(23.1%)  14(22.2%)  38(33.3%)  13(10.8%) |
| 1. Pai 2024   (NCT02891226) | Induction  12 weeks | Mirikizumab 200 mg IV (65)  Mirikizumab 600 mg IV (63)  Mirikizumab 1000 mg IV (115)  Placebo (118) | NR | NR | NR | NR | NR | NR | 34(52.3%)  30(47.6%)  78(67.8%)  38(32.2%) | 15(23.1%)  14(22.2%)  39(33.9%)  12(10.2%) |
| 1. E. Sands 2017   (NCT01714726) | Induction  12 weeks | MEDI2070 700 mg IV (59)  Placebo (60) | 22(37.3%)  17(28.3%) | NR | 12(20.3%)  8(13.3%) | NR | NR | NR | NR | NR |
| 1. Sandborn 2022   (NCT03466411) | Induction  12 weeks | Guselkumab 200 mg IV (61)  Guselkumab 600 mg IV (63)  Guselkumab 1200 mg IV (61)  Placebo (61) | 43(70.5%)  42(66.7%)  37(60.7%)  15(24.6%) | NR | 35(57.4%)  35(55.6%)  28(45.9%)  10(16.4%) | 27(44.3%)  32(50.8%)  20(32.8%)  10(16.4%) | 23(37.7%)  23(36.5%)  20(32.8%)  7(11.5%) | NR | NR | NR |
| 1. Danese 2024   (NCT03466411) | Maintenance  48 weeks | Guselkumab 200→100 mg IV→SC (61)  Guselkumab 600→200 mg IV→SC (63)  Guselkumab 1200→200 mg IV→SC (61)  Placebo (15) | NR | NR | 39(64%)  46(73%)  35(57%)  9(60%) | NR | 27(44%)  29(46%)  27(44%)  2(13%) | NR | NR | NR |
| 1. D’Haens 2021 | Induction  12 weeks | Guselkumab 200 mg IV (50)  Guselkumab 600 mg IV (50)  Guselkumab 1200 mg IV (50)  Placebo (51) | NR | NR | NR | NR | 18(36%)  20(40%)  18(36%)  6(11.8%) | 8(16%)  5(10%)  8(16%)  2(3.9%) | NR | NR |

**Supplementary Table 4.** Summary of clinical, endoscopic, and histologic efficacy outcomes of CD.

| Study reference | Therapy period & time to primary outcome | Treatment Arms (n) | Clinical Response n (%) | Clinical Remission n (%) | Endoscopic Response n (%) | Endoscopic Remission n (%) | Histologic Response n (%) | Histologic Remission n (%) |
| --- | --- | --- | --- | --- | --- | --- | --- | --- |
| 1. Louis 2023   (NCT03398148) | Induction  12 weeks | Risankizumab 1200 mg IV (650)  Placebo (325) | 418(64.3%)  116(35.7%) | 132(20.3%)  20(6.2%) | 237(36.5%)  39(12.1%) | 69(10.6%)  11(3.4%) | 159(24.5%)  25(7.7%) | 41(6.3%)  2(0.6%) |
| 1. Sandborn 2020   (NCT02589665) | Induction  12 weeks | Mirikizumab 50 mg IV (63)  Mirikizumab 200 mg IV (62)  Mirikizumab 600 mg IV (61)  Placebo (63) | 26(41.3%)  37(59.7%)  30(49.2%)  13(20.6%) | 10(15.9%)  14(22.6%)  7(11.5%)  3(4.8%) | 15(23.8%)  19(30.6%)  8(13.1%)  4(6.3%) | 2(3.2%)  2(3.2%)  1(1.6%)  1(1.6%) | NR | 9(14.3%)  28(45.2%)  21(34.4%)  11(17.5%) |
| 1. Sandborn 2020   (NCT02589665) | Maintenance  40 weeks | Mirikizumab 200 mg SC Q4W (47)  Mirikizumab 200 mg SC Q12W (46)  Placebo (13) | 38(80.9%)  35(76.1%)  7(53.8%) | 22(46.8%)  17(37.0%)  1(7.7%) | 27(57.4%)  22(47.8%)  2(15.4%) | 7(14.9%)  13(28.3%)  1(7.7%) | NR | 31(66.0%)  17(37.0%)  5(38.5%) |
| 1. D’Haens 2023   (NCT03518086) | Induction  12 weeks | Mirikizumab 300mg IV (868)  Placebo (294) | 551(63.5%)  124(42.2%) | 210(24.2%)  39(13.3%) | 315(36.3%)  62(21.1%) | NR | 235(27.1%)  41(13.9%) | NR |
| 1. D’Haens 2023   (NCT03524092) | Maintenance  40 weeks | Mirikizumab 200mg SC (365)  Placebo (179) | NR | 182(49.9%)  45(25.1%) | 214(58.6%)  52(29.1%) | NR | 158(43.3%)  39(21.8%) | NR |
| 1. Magro 2023   (NCT03518086) | Induction  12 weeks | Mirikizumab 300mg IV (868)  Placebo (294) | NR | NR | 315(36.3%)  62(21.1%) | NR | 340(39.2%)  61(20.7%) | 254(29.3%)  46(11.5%) |
| 1. Magro 2023   (NCT03524092) | Maintenance  40 weeks | Mirikizumab 200mg SC (365)  Placebo (179) | NR | NR | 214(58.6%)  52(29.1%) | NR | 200(54.8%)  46(25.7%) | 177(48.5%)  44(24.6%) |
| 1. Kobayashi 2024   (NCT03518086) | Induction  12 weeks | Mirikizumab 300mg IV (102)  Placebo (35) | 73(71.6%)  8(22.9%) | 33(32.4%)  1(2.9%) | 42(41.2%)  3(8.6%) | NR | 33(32.4%)  2(5.7%) | NR |
| 1. Kobayashi 2024   (NCT03524092) | Maintenance  40 weeks | Mirikizumab 200mg SC (47)  Placebo (25) | 23(48.9%)  7(28.0%) | NR | 27(57.4%)  7(28.0%) | NR | 23(48.9%)  624.0%) | NR |
| 1. Peyrin-Biroulet 2023   (NCT04033445) | Induction  12 weeks | Guselkumab 200 mg IV (101)  Guselkumab 400 mg IV (107)  Placebo (105) | 62(61.4%)  65(60.7%)  29(27.6%) | 26(25.7%)  27(25.2%)  10(9.5%) | 31(30.7%)  33(30.8%)  13(12.4%) | 18(17.8%)  15(14.0%)  7(6.7%) | 21(20.8%)  29(27.1%)  9(8.6%) | NR |

**Supplementary Table 5.** Summary of clinical, endoscopic, and histologic efficacy outcomes of UC.
